# Supplementary material for: The effect of cold ischemia time on hypoxia, EMT, and apoptosis pathways in normal colon mucosa
Source: PLoS One. 2026 Mar 20;21(3):e0321432. doi: 10.1371/journal.pone.0321432 (PMC13004397; doi:10.1371/journal.pone.0321432)
Supplement: Attachment — Submitted filename: PONE-D-25-11860_R1.pdf [file pone.0321432.s003.pdf]

PLOS ONE

The effect of cold ischemia time on hypoxia, EMT, and apoptosis pathways in normal colon mucosa

--Manuscript Draft--

**Manuscript Number:**	PONE-D-25-11860R1
**Article Type:**	Research Article
**Full Title:**	The effect of cold ischemia time on hypoxia, EMT, and apoptosis pathways in normal colon mucosa
**Short Title:**	Cold ischemia time influences transcriptional landscape of colon mucosa
**Corresponding Author:**	Natalia Filipowicz, Ph.D.<br>Medical University of Gdansk: Gdanski Uniwersytet Medyczny<br>Gdansk, POLAND
**Keywords:**	cold ischemia, hypoxia, post-zygotic mutations, preanalytical variable, epithelial-to-mesenchymal transition (EMT), colon mucosa, non-tumorous tissue, Weighted Gene Co-expression Network Analysis (WGCNA)
**Abstract:**	Cold ischemia time (CIT), the interval between tissue excision and preservation, is a critical preanalytical variable that profoundly impacts gene expression profiles. Variability in CIT can lead to inconsistent transcriptomic results, making study interpretation challenging and undermining reproducibility in biomedical research. Our study aimed to evaluate the impact of CIT on the expression of cancer-related genes, particularly these involved in hypoxia, apoptosis, and epithelial-to-mesenchymal transition (EMT). We performed RNA sequencing on 54 normal colon mucosa samples from nine patients undergoing colorectal cancer surgeries, freezing samples at predefined intervals ranging from 0 to 60 minutes. A total of 44 differentially expressed genes (DEGs) ($p < 0.05$) were identified when comparing samples frozen immediately (T0) with those frozen after 60 minutes (T5). These DEGs were further analyzed through functional and pathway enrichment analyses and weighted gene co-expression network analysis (WGCNA). The enrichment analysis revealed significant alterations in pathways associated with apoptosis, hypoxia, EMT, and cancer progression, including p53 and HIF-1 signaling. WGCNA highlighted two co-expressed gene modules: ME2, which showed downregulation of apoptosis-related genes, and ME4, linked to apoptosis and cellular metabolism. Our findings highlight CIT as a critical preanalytical variable, showing that prolonged ischemia can induce transcriptomic changes that may mimic malignancy, and potentially confound research outcomes. To minimize such effects, we recommend keeping CIT under 45–60 minutes.
**Order of Authors:**	Katarzyna Duzowska, MPharm
	Natalia Filipowicz, PhD
	Mikołaj Opiełka, MD
	Kinga Drężek-Chyła, MSc
	Anna Kostecka, PhD
	Monika Horbacz, MSc
	Jarosław Skokowski, PhD
	Olga Rostkowska, MD
	Jarosław Kobiela, PhD
	Leszek Kalinowski, PhD
	Jan P. Dumanski, PhD
	Arkadiusz Piotrowski, PhD
	Marcin Jąkalski, PhD

Powered by Editorial Manager® and ProduXion Manager® from Aries Systems Corporation

Response to Reviewers:	1. Is the manuscript technically sound, and does the data support the conclusions?<br>The manuscript must describe a technically sound piece of scientific research with data that supports the conclusions. Experiments must have been conducted rigorously, with appropriate controls, replication, and sample sizes. The conclusions must be drawn appropriately based on the data presented.<br>Reviewer #1: Yes<br>Reviewer #2: Yes<br><br>2. Has the statistical analysis been performed appropriately and rigorously?<br>Reviewer #1: Yes<br>Reviewer #2: Yes<br><br>3. Have the authors made all data underlying the findings in their manuscript fully available?<br>The PLOS Data policy requires authors to make all data underlying the findings described in their manuscript fully available without restriction, with rare exception (please refer to the Data Availability Statement in the manuscript PDF file). The data should be provided as part of the manuscript or its supporting information, or deposited to a public repository. For example, in addition to summary statistics, the data points behind means, medians and variance measures should be available. If there are restrictions on publicly sharing data—e.g. participant privacy or use of data from a third party—those must be specified.<br>Reviewer #1: Yes<br>Reviewer #2: Yes<br><br>4. Is the manuscript presented in an intelligible fashion and written in standard English?<br>PLOS ONE does not copyedit accepted manuscripts, so the language in submitted articles must be clear, correct, and unambiguous. Any typographical or grammatical errors should be corrected at revision, so please note any specific errors here.<br>Reviewer #1: Yes<br>Reviewer #2: Yes<br>5. Review Comments to the Author<br>Please use the space provided to explain your answers to the questions above. You may also include additional comments for the author, including concerns about dual publication, research ethics, or publication ethics. (Please upload your review as an attachment if it exceeds 20,000 characters)<br>Reviewer #1: accepted.<br>explain your answers to the questions above. You may also include additional comments for the author, including concerns about dual publication, research ethics, or publication ethics. (Please upload your review as an attachment if it exceeds 20,000 characters) (Limit 200 to 20000 Characters)<br>Reviewer #2: The paper explains the effect of cold ischemia time (CIT) and its importance in transcriptomic studies and reproducibility. The study objective evaluating CIT effects on cancer-related genes is relevant and practical. The findings from this study suggest a CIT limit (≤45–60 min), information that is valuable for the research community.<br>The number of samples, patient count and CIT intervals are clearly described but whether all 54 samples were paired per patient across all time points needs further clarification.<br>Answer: All 54 samples used in our study originate from 9 CRC patients, with samples collected at 6 distinct time points as defined in the manuscript (9 patients × 6 time points = 54 samples). Thus, each patient contributed one sample per time point, resulting in a fully paired dataset.<br>We accounted for the paired nature of the RNAseq data by treating the patient ID as batch and removing its effect from the data with ComBat-seq. The sentence clarifying this was modified in lines 173-175.<br>Overall, the analysis is comprehensive. The combination of RNA-seq, functional enrichment and WGCNA strengthens the biological interpretation. Identifying DEGs and linking them to apoptosis, hypoxia, EMT and cancer-related pathways is relevant and informative.<br><br>6. PLOS authors have the option to publish the peer review history of their article (what does this mean?). If published, this will include your full peer review and any attached

	files.<br>If you choose "no", your identity will remain anonymous but your review may still be made public.<br>Do you want your identity to be public for this peer review? For information about this choice, including consent withdrawal, please see our Privacy Policy.<br>Reviewer #1: No<br>Reviewer #2: No
Additional Information:	
Question	Response
**Financial Disclosure**<br><br>Enter a financial disclosure statement that describes the sources of funding for the work included in this submission. Review the submission guidelines for detailed requirements. View published research articles from *PLOS ONE* for specific examples.<br><br>This statement is required for submission and **will appear in the published article** if the submission is accepted. Please make sure it is accurate.<br><br>**Funded studies**<br>Enter a statement with the following details:<br>• Initials of the authors who received each award<br>• Grant numbers awarded to each author<br>• The full name of each funder<br>• URL of each funder website<br>• Did the sponsors or funders play any role in the study design, data collection and analysis, decision to publish, or preparation of the manuscript?<br><br>Did you receive funding for this work?	Yes
Please add funding details.<br>as follow-up to "**Financial Disclosure**<br><br>Enter a financial disclosure statement that describes the sources of funding for the work included in this submission. Review the submission guidelines for detailed requirements. View published research articles from *PLOS ONE* for specific examples.	This study was supported by the Foundation for Polish Science (FNP) under the International Research Agendas Program to J.P.D. and A.P., co-financed by the European Union under the European Regional Development Fund (MAB/2018/6); and the Ministry of Science and Higher Education, (grant No. 2/566516/ SPUB/SP/2023) to L.K.

This statement is required for submission and **will appear in the published article** if the submission is accepted. Please make sure it is accurate.<br><br>**Funded studies**<br>Enter a statement with the following details:<br>• Initials of the authors who received each award<br>• Grant numbers awarded to each author<br>• The full name of each funder<br>• URL of each funder website<br>• Did the sponsors or funders play any role in the study design, data collection and analysis, decision to publish, or preparation of the manuscript?<br><br>Did you receive funding for this work?"	
Please select the country of your main research funder (please select carefully as in some cases this is used in fee calculation).<br>as follow-up to "**Financial Disclosure**<br><br>Enter a financial disclosure statement that describes the sources of funding for the work included in this submission. Review the submission guidelines for detailed requirements. View published research articles from *PLOS ONE* for specific examples.<br><br>This statement is required for submission and **will appear in the published article** if the submission is accepted. Please make sure it is accurate.<br><br>**Funded studies**<br>Enter a statement with the following details:<br>• Initials of the authors who received each award<br>• Grant numbers awarded to each author<br>• The full name of each funder<br>• URL of each funder website<br>• Did the sponsors or funders play any role in the study design, data collection and analysis, decision to publish, or preparation of the manuscript?	POLAND - PL

Did you receive funding for this work?"	
**Competing Interests**<br><br>Use the instructions below to enter a competing interest statement for this submission. On behalf of all authors, disclose any competing interests that could be perceived to bias this work—acknowledging all financial support and any other relevant financial or non-financial competing interests.<br><br>This statement is required for submission and **will appear in the published article** if the submission is accepted. Please make sure it is accurate and that any funding sources listed in your Funding Information later in the submission form are also declared in your Financial Disclosure statement.<br><br>View published research articles from *PLOS ONE* for specific examples.<br><br>**NO authors have competing interests**<br><br>Enter: *The authors have declared that no competing interests exist.*<br><br>**Authors with competing interests**<br><br>Enter competing interest details beginning with this statement:<br><br>*I have read the journal's policy and the authors of this manuscript have the following competing interests: [insert competing interests here]*<br><br>* typeset	J.P.D. is cofounder and shareholder in Cray Innovation AB. The remaining authors have declared no competing interests.
**Ethics Statement**<br><br>Enter an ethics statement for this submission. This statement is required if the study involved:	All procedures for sample collection were approved by the Independent Bioethics Committee for Research at the Medical University of Gdansk (approval number NKBBN/564/2018 with multiple amendments). Written informed consent was obtained from all the patients prior to surgery. All procedures were performed in accordance with the relevant national and international laws and guidelines as well as in compliance

• Human participants<br>• Human specimens or tissue<br>• Vertebrate animals or cephalopods<br>• Vertebrate embryos or tissues<br>• Field research<br><br>Write "N/A" if the submission does not require an ethics statement.<br><br>General guidance is provided below. Consult the submission guidelines for detailed instructions. **Make sure that all information entered here is included in the Methods section of the manuscript.**	with European Union General Data Protection Regulation (EU GDPR).

**Format for specific study types**<br><br>**Human Subject Research (involving human participants and/or tissue)**<br>• Give the name of the institutional review board or ethics committee that approved the study<br>• Include the approval number and/or a statement indicating approval of this research<br>• Indicate the form of consent obtained (written/oral) or the reason that consent was not obtained (e.g. the data were analyzed anonymously)<br><br>**Animal Research (involving vertebrate animals, embryos or tissues)**<br>• Provide the name of the Institutional Animal Care and Use Committee (IACUC) or other relevant ethics board that reviewed the study protocol, and indicate whether they approved this research or granted a formal waiver of ethical approval<br>• Include an approval number if one was obtained<br>• If the study involved *non-human primates*, add *additional details* about animal welfare and steps taken to ameliorate suffering<br>• If anesthesia, euthanasia, or any kind of animal sacrifice is part of the study, include briefly which substances and/or methods were applied<br><br>**Field Research**<br><br>Include the following details if this study involves the collection of plant, animal, or other materials from a natural setting:<br>• Field permit number<br>• Name of the institution or relevant body that granted permission	
**Data Availability**<br><br>Authors are required to make all data underlying the findings described fully available, without restriction, and from the time of publication. PLOS allows rare exceptions to address legal and ethical concerns. See the PLOS Data Policy and FAQ for detailed information.	Yes - all data are fully available without restriction

A Data Availability Statement describing where the data can be found is required at submission. Your answers to this question constitute the Data Availability Statement and **will be published in the article**, if accepted.<br><br>**Important:** Stating 'data available on request from the author' is not sufficient. If your data are only available upon request, select 'No' for the first question and explain your exceptional situation in the text box.<br><br>Do the authors confirm that all data underlying the findings described in their manuscript are fully available without restriction?	
**Describe where the data may be found in full sentences. If you are copying our sample text, replace any instances of XXX with the appropriate details.**<br><br>• If the data are **held or will be held in a public repository**, include URLs, accession numbers or DOIs. If this information will only be available after acceptance, indicate this by ticking the box below. For example: *All XXX files are available from the XXX database (accession number(s) XXX, XXX.*).<br>• If the data are all contained **within the manuscript and/or Supporting Information files**, enter the following: *All relevant data are within the manuscript and its Supporting Information files.*<br>• If neither of these applies but you are able to provide **details of access elsewhere**, with or without limitations, please do so. For example:<br><br>*Data cannot be shared publicly because of [XXX]. Data are available from the XXX Institutional Data Access / Ethics Committee (contact via XXX) for researchers who meet the criteria for access to confidential data.*<br><br>*The data underlying the results presented in the study are available from (include the name of the third party*	The 54 files of raw data from RNAseq are available from European Genome-phenome Archive (EGA) under the accession number EGAD00001015661 under request.

Powered by Editorial Manager® and ProduXion Manager® from Aries Systems Corporation

and contact information or URL).<br>• This text is appropriate if the data are owned by a third party and authors do not have permission to share the data.<br><br>* typeset	
Additional data availability information:	Tick here if the URLs/accession numbers/DOIs will be available only after acceptance of the manuscript for publication so that we can ensure their inclusion before publication.

Powered by Editorial Manager® and ProduXion Manager® from Aries Systems Corporation

Response to Reviewers

Click here to access/download;Manuscript;PONED2511860_Duzowska_et_

The effect of cold ischemia time on hypoxia, EMT, and apoptosis pathways in normal colon mucosa

Katarzyna Duzowska[1], Mikołaj Opiełka[1,2], Kinga Drężek-Chyła[1], Anna Kostecka[1], Monika Horbacz[1], Jarosław Skokowski[3], Olga Rostkowska[4], Jarosław Kobiela[4], Leszek Kalinowski[5,6], Jan P. Dumanski[1,7], Arkadiusz Piotrowski[1], Marcin Jąkalski[1,8&]*, Natalia Filipowicz[1&]*

[1] 3P-Medicine Laboratory, Medical University of Gdańsk, Gdańsk, Poland

[2] Department of Biochemistry, Medical University of Gdańsk, Gdańsk, Poland

[3] Academy of Applied Medical and Social Science, Elbląg, Poland

[4] Department of Oncological, Transplant and General Surgery, Medical University of Gdańsk, Gdańsk, Poland

[5] Department of Medical Laboratory Diagnostics, Medical University of Gdańsk, Gdańsk, Poland

[6] BioTechMed Center, Department of Mechanics of Materials and Structures, Gdańsk University of Technology, Gdańsk, Poland

[7] Department of Immunology, Genetics and Pathology and Science for Life Laboratory, Uppsala University, Uppsala, Sweden

[8] Center for Applied Genomics and Bioinformatics, Faculty of Biology, University of Gdańsk, Gdańsk, Poland

[&]these authors contributed equally to this work

* Corresponding author:

Email: natalia.filipowicz@gumed.edu.pl and marcin.jakalski@ug.edu.pl

Field Code Changed

Field Code Changed

Short title

Cold ischemia time influences transcriptional landscape of colon mucosa

Keywords

cold ischemia, hypoxia, post-zygotic mutations, preanalytical variable, epithelial-to-mesenchymal transition (EMT), targeted RNA sequencing, colon mucosa, non-tumorous tissue, differentially expressed genes, weighted gene co-expression network analysis (WGCNA)

Abstract

Cold ischemia time (CIT), the interval between tissue excision and preservation, is a critical preanalytical variable that profoundly impacts gene expression profiles. Variability in CIT can lead to inconsistent transcriptomic results, making study interpretation challenging and undermining reproducibility in biomedical research. Our study aimed to evaluate the impact of CIT on the expression of cancer-related genes, particularly these involved in hypoxia, apoptosis, and epithelial-to-mesenchymal transition (EMT). We performed RNA sequencing on 54 normal colon mucosa samples from nine patients undergoing colorectal cancer surgeries, freezing samples at predefined intervals ranging from 0 to 60 minutes. A total of 44 differentially expressed genes (DEGs) ($p < 0.05$) were identified when comparing samples frozen immediately (T0) with those frozen after 60 minutes (T5). These DEGs were further analyzed through functional and pathway enrichment analyses and weighted gene co-expression network analysis (WGCNA). The enrichment analysis revealed significant alterations in pathways associated with apoptosis, hypoxia, EMT, and cancer progression,

including p53 and HIF-1 signaling. WGCNA highlighted two co-expressed gene modules: ME2, which showed downregulation of apoptosis-related genes, and ME4, linked to apoptosis and cellular metabolism. Our findings highlight CIT as a critical preanalytical variable, showing that prolonged ischemia can induce transcriptomic changes that may mimic malignancy, and potentially confound research outcomes. To minimize such effects, we recommend keeping CIT under 45–60 minutes.

Introduction

The quality and integrity of biological samples are crucial for multi-omic studies, directly impacting the reliability of results. Preanalytical variables, including patient-specific factors and tissue handling protocols, can significantly impact the outcomes (1,2). With biobanking playing a crucial role in biomedical translational research, standardized tissue collection protocols are essential to minimize external variability (3–5). Ischemia, defined as a restriction of blood supply to tissues or organs, results in a shortage of oxygen supply necessary to sustain the cellular metabolism. In the *ex vivo* context, this process can be classified into warm and cold ischemia. Cold ischemia time (CIT) refers to the condition in which oxygen supply is entirely absent after tissue excision while the sample is maintained at temperatures below body temperature (6). Warm ischemia time (WIT) refers to the period during which the tissue remains in the donor's body but the oxygen supply is insufficient to meet metabolic demands. This preanalytical variable is challenging to control as it relies on the efficiency of the surgical team, for which the priority is the successful completion of the operation. Reports on the impact of WIT on transcriptomic profiles are scarce, but studies, including those by Pedersen *et al.* and Ma *et al.*, highlight its effects, identifying numerous differentially expressed genes (DEGs) and alterations in oncogenic, inflammatory, and immunological

pathways (7,8). In contrast, CIT is determined by the biobanking procedure and can be effectively managed by the tissue collector (9). Prolonged CIT, rather than malignancy, can lead to specific, even subtle, changes in gene expression profiles, complicating the interpretation of tissue samples.

Several studies have assessed the impact of CIT on RNA quality, measured as RNA integrity number (RIN), reporting minimal changes up to 4 hours (10–12) and even up to 16 hours at room temperature (13). RNA quality also varies by tissue type, with thyroid and colorectal tissues being more sensitive to cold ischemia compared to stomach and lung tissues (14). Gastrointestinal samples, in general, tend to exhibit lower RNA quality than those from other organs (2,15), with tumor tissues displaying significantly higher RINs than normal tissues, though CIT has little effect on this observed difference (2).

While most reports focus on RNA integrity, few have explored the impact of CIT on gene expression, particularly in cancer-related pathways (11,16,17). Transcriptomic analyses suggest that CIT exceeding 60 minutes can significantly alter gene expression. Aktas *et al*. identified 41 transcripts affected by prolonged CIT, including apoptosis- and cell cycle-related genes, with over 3% showing significant changes (10). Other study reported transcriptomic alterations as early as 30 minutes post-excision, for instance delayed freezing of colorectal carcinoma samples lead to increased KLF6 expression, potentially misrepresenting its role in tumor pathogenesis (16). A study of transcriptomic profiles in renal carcinoma showed over 4,000 genes were affected at longer CIT durations and higher temperatures, emphasizing the need for immediate snap-freezing to preserve gene expression profiles relevant to cancer research (11).

Despite these findings, the existing literature remains limited, particularly regarding the effect of cold ischemia time (CIT) on the transcription of cancer-associated genes, including those involved in epithelial-to-mesenchymal transition (EMT), hypoxia, apoptosis, and cell death. These genes are crucial for understanding cancer biology and the complex interactions within the tumor microenvironment (18). Moreover, their expression can directly influence treatment decisions and patient outcomes (16,18–20). Some of these genes may be first responders to cold ischemia, making them potential markers of CIT. Examining these preanalytical factors is essential not only to enhance the reliability and reproducibility of experimental findings but also to ensure that scientific conclusions translate effectively into clinical practice.

The primary aim of this research is to investigate the influence of CIT on the transcription profile of macro- and microscopically normal colon mucosa collected from patients with colorectal cancer. The focus on normal colon mucosa aligns with the design of ongoing research in our laboratory, which investigates genetic mosaicism focusing on post-zygotic mutations accumulating in morphologically normal tissues proximal and distal to tumors (4,21). Furthermore, the majority of the publicly available studies focused on tumor tissue, with limited insights into healthy tissue (2,13,17). Here we employed targeted RNA sequencing (NGS RNAseq), including, among the others, genes associated with hypoxia, apoptosis, and cancer, thus addressing a critical gap in the literature on preanalytical variables for tissue collection and subsequent analysis for cancer research.

Materials and methods

Study Design and Sample Handling

The healthy colon mucosa samples were collected from 9 consecutive patients (2 females; 7 males; mean age 68 years ± 10.9) diagnosed with colorectal cancer who underwent surgical resection 21st of April to 24th of August 2021 in the Department of Oncological, Transplant, and General Surgery; University Clinical Center in Gdansk. The summary of patients' data is presented in Table 1. After the surgical resection, a larger, full-walled specimen of the colon was immediately excised approximately 10 - 15 cm away from the primary tumor, rinsed in saline and left at room temperature until the mucosa collection procedure at a particular time point was completed. At each time point, a sample of macroscopically unaffected mucosa was excised by dissecting from the colon specimen and detaching gently from the muscular layer. Subsequent washing steps were implemented, including: saline solution (twice), antibiotic solution (Penicillin - Streptomycin 5000 U/ml), and saline solution, followed by snap freezing in liquid nitrogen. Tissue fragments were collected at six time points - 0 (T0), 10 (T1), 20 (T2), 30 (T3), 45 (T4), and 60 (T5) minutes after the surgical resection, estimated as the time of cold ischemia. Sample collected at T0 was processed immediately after organ resection. First time point (T0) was used as the reference and the starting point for all the subsequent analyses. In total 54 samples were collected and subjected to further analysis.

RNA Extraction and Quality Control

Colon mucosa samples were stored at -80°C until RNA isolation. Total RNA was extracted from 10 - 30 mg of tissue samples that were mechanically homogenized using T10 Basic ULTRA - TURRAX disperser (IKA) in the presence of QIAzol Lysis reagent (Qiagen). RNA isolation, purification, and DNase digestion were performed using the RNeasy Mini kit (Qiagen) according to the original protocol with two modifications as described in the previous paper (22) RNA quality and quantity were assessed using TapeStation (Agilent Technologies) using

RNA ScreenTape kit according to the manufacturer's protocol (Table 2). RNA integrity number (RIN) was calculated using TapeStation analysis software (Agilent Technologies). Additionally, the DV200 index, indicating the percentage of RNA fragments > 200 nt, was calculated and used as a quality assessment standard (23). Samples with a DV200 index > 75% were used for further NGS analysis.

Targeted RNA sequencing

The targeted RNA sequencing panel was designed with the Roche NimbleDesign online tool (Roche, now HyperDesign, https://hyperdesign.com/#/) and covered 7229 regions with a total length of 1,243,523 bp. It included 634 genes based on literature research and covered genes associated with apoptosis, cell death, hypoxia, epithelial-to-mesenchymal transition, and other cancer-related genes (the full list of transcripts is given in S2 Table). NGS libraries were prepared with the Kapa RNA HyperPrep Kit (Roche) using Automated Liquid Handling Bravo NGS workstation (Agilent Technologies) according to the manufacturer manual with 100 ng RNA used as an input and enzymatic fragmentation at 94°C for 6 minutes, with the addition of ERCC RNA Spike-In Mix (Invitrogen) as an external RNA control. All the single libraries were multiplexed and hybridized with SeqCap EZ Choice Probes (Roche) designed by our group and KAPA HyperCapture Reagent and Bead kit (v2, Roche Sequencing Solutions, Inc.) according to SeqCap RNA Enrichment System User's Guide (v.1.0) with slight modifications. Component A was replaced with formaldehyde, and the Multiplex Hybridization Enhancing Oligo Pool was replaced with Universal Blocking Oligos (UBO).The hybridization was run for 18h, the library was cleaned up, post-captured, amplified, and purified, followed by inspection of fragment distribution using TapeStation with High Sensitivity D1000 Screen Tape kit (Agilent Technologies) and qPCR quantitation in a Roche

Field Code Changed

Field Code Changed

LightCycler 480 with KAPA Library Quantification Kit (Roche). Paired-end reads of 150 bp were generated using TruSeq RNA Access sequencing chemistry by an external service provider (Macrogen Europe, Amsterdam, The Netherlands).

Transcriptomic data analysis

The RNA-seq data were processed as described in Andreou et al. 2024 (24). Briefly, after the quality filtering and trimming of the raw FASTQ files with BBDuk (https://sourceforge.net/projects/bbmap/, version 38.36), the resulting reads were mapped to the reference human genome (hg38, GENCODE version 39) using STAR version 2.7.3a (25). Raw read counts assigned to the annotated genes obtained in the above process were collated into a single gene expression matrix and processed further in R programming language (https://www.r-project.org, version 4.1.2). Lowly expressed genes were filtered out based on a minimal required CPM expression threshold. The filtered gene expression matrix was normalized using the TMM method in edgeR (26). Principal Component Analysis (PCA) was performed to investigate sample grouping and identify potential outliers using FactoMineR, version 2.4 (27). We accounted for the paired nature of the RNAseq data by treating the patient ID as batch and removing its effect from the data using ComBat-seq (28). Significantly differentially expressed genes (DEGs) were identified with EdgeR using the glmLRT function (likelihood ratio test) with a significance threshold set to 0.05 (False Discovery Rate, FDR).

Weighted gene co-expression network analysis (WGCNA) was performed on the normalized and batch-adjusted gene expression values using the WGCNA R library WGCNA (29). Soft threshold value for building the correlation network was selected empirically based on diagnostic plots (pickSoftThreshold function). The final network was built using the

blockwiseModules function, where the TOMType parameter was set to “signed”. Subsequently, a linear model (lmFit, from the limma package, (30)) was run on all modules to identify those associated with the tested trait (time point).

Overrepresented terms / GO / pathways

Overrepresentation analysis (ORA) was used to perform functional enrichment analysis of significantly differentially expressed genes, utilizing the Gene Ontology (GO) database and the genome reference set in WebGestalt (31). Pathway enrichment analysis was similarly conducted with ORA, focusing on KEGG pathways and the genome reference set in WebGestalt. In addition, functional and pathway enrichment analyses were separately applied to genes from WGCNA modules ME2 and ME4, using WebGestalt for the analysis.

Bioethics Committee Approval

All procedures for sample collection were approved by the Independent Bioethics Committee for Research at the Medical University of Gdansk (approval number NKBBN/564/2018 with multiple amendments). Written informed consent was obtained from all the patients prior to surgery. All procedures were performed in accordance with the relevant national and international laws and guidelines as well as in compliance with European Union General Data Protection Regulation (EU GDPR).

Table 1. A summary of donors and diagnoses included in the study

Patient ID	***Sex***	***Age***	***ICD-10 Classification***
ES31C	Male	67	C18.7 - Malignant neoplasm of sigmoid colon
5IDSR	Female	70	C19 - Malignant neoplasm of rectosigmoid junction
OV1IW	Male	67	C20 - Malignant neoplasm of rectum

NYYCH	Male	48	C20 - Malignant neoplasm of rectum
LZZOP	Female	72	C19 - Malignant neoplasm of rectosigmoid junction
JSDHP	Male	68	C19 - Malignant neoplasm of rectosigmoid junction
PZ94B	Male	83	C18.7 - Malignant neoplasm of sigmoid colon
FFMB1	Male	59	C20 - Malignant neoplasm of rectum
ABCD	Male	83	C18.5 - Malignant neoplasm of splenic flexure

Results

Quality of total RNA within the studied time frame

Tissues from nine patients were collected and snap-frozen at six time points: immediately post-resection (T0), 10 minutes (T1), 20 minutes (T2), 30 minutes (T3), 45 minutes (T4), and 60 minutes (T5) post-resection (see Materials and Methods). As many prior studies have focused on RNA quality as a consequence of CIT, we performed quality control by measuring both RIN and DV200 values. Notably, RIN values remained consistent across all analyzed time points, with a median value of 4.3. Furthermore, all samples had DV200 above 75%, indicating that RNA was of sufficient quality for subsequent analysis (32). The consistency of these results across all time points suggest that RNA integrity was preserved throughout the collection and processing protocol. Detailed RIN measurements for each sample are provided in S1 Table.

Differential Expression Analysis highlights genes associated with hypoxia, EMT, and apoptosis

Differential expression analysis revealed 44 significantly differentially expressed genes (DEGs) between the two extreme time points set for our study, T0 and T5 (60 min) (S3 Table). Principal component analysis (PCA) demonstrated a noticeable separation between samples at T0 and T5 after eliminating patient batch effect, as illustrated in Fig. 1. Comparisons involving T0 and other time points (T1–T4) showed fewer statistically significant DEGs: zero between T0 vs T3, and six for T0 vs T4, namely ABCB1, ABCG2, EPAS1, FZD2, LAMA3, and TMPRSS2. Our results show that the initial gene expression changes occur between 30 minutes (T3) and 45 minutes (T4) post-excision; with 44 genes displaying significant changes between 45 minutes and 60 minutes (T5) after excision (S3 Table). Thus, our further analyses focused on the T0 versus T5 comparison.

Applying an absolute logFC cutoff of ≥1, we identified 15 upregulated genes and 29 downregulated genes in T5 compared to T0. Notably, DEGs included hypoxia- and EMT-associated genes such as SNAI, CDH1, ADM, MMP9, EGLN3, IL-6, AKT3, TSPAN1, and DSC2, as well as apoptosis-related genes: CASP7, TNFSF10, DEFB1, AKT3, and JPH3. Among the 44 DEGs identified between T0 and T5 (S3 Table), 5 were linked to hypoxia, 8 to apoptosis or cell death, and 10 to epithelial-to-mesenchymal transition (EMT) determined using EMTome (33). The remaining 19 were classified as 'other'. It is important to stress that none of 22 housekeeping genes used as a reference set included in the panel showed significant expression changes. S3 Table provides the complete DEG list with the logFC, p-values, and FDR values.

Functional enrichment of DEGs using overrepresentation analysis (ORA) identified enrichment of Gene Ontology (GO) terms associated with processes such as apoptosis, cell-cell adhesion, tissue migration, and others. TNF signaling, ABC transporters, gastric cancer, and cancer pathways were among the enriched pathways shown by ORA KEGG analysis (S4 Table).

Fig. 1: Principal component analysis (PCA) of all 54 samples using the normalized batch-corrected expression profiles of 1419 genes (both targeted and non-targeted). Each point represents the orientation of a sample projected into the transcriptional space, color and symbol refer to its group membership. The analysis demonstrated clearly separated clusters of T0 and T5 samples (marked in green and yellow circles), a pattern not pronounced among the intermediate time points (T1-T4). T0 - time point 0, frozen immediately after resection; T1 - time point 1, frozen 10 minutes after resection; T2 - time point 2, frozen 20 minutes after resection; T3 - time point 3, frozen 30 minutes after resection; T4 - time point 4, frozen 45 minutes after resection; T5 - time point 5, frozen 60 minutes after resection.

Weighted Gene Co-Expression Network Analysis (WGCNA) confirms two gene modules associated with apoptosis, EMT and hypoxia

To find the clusters of highly correlated genes in our dataset, we applied the WGCNA analysis that identified two significant gene modules, ME2 and ME4 (adjusted p-values 0.037 and 0.018, respectively), which demonstrate distinct patterns during the time course of our study. Notably, the genes within these modules were associated with key biological processes, including apoptosis, EMT, and hypoxia-related pathways (Fig. 2).

ME2 consisted of 145 genes, primarily exhibiting a downregulation pattern in T5 in relation to T0 (Fig. 2A, 2B). Among these, 26.2% were related to apoptosis or cell death, 18.6% to EMT, 11.7% to hypoxia, and 0.69% (one gene) was annotated as a reference gene in our targeted panel (Fig. 2C). The remaining 42.8% were categorized as 'other' (see details in S5 Table). Functional enrichment analysis of ME2 genes using ORA GO (FDR < 0.05) identified several pathways, including those related to the execution phase of apoptosis and the extrinsic apoptotic signaling pathway. KEGG pathway analysis further highlighted nine pathways,

including cancer-related and apoptosis-related processes such as p53 signaling, HIF-1 signaling, and FoxO signaling (S7 Table).

ME4, a smaller gene module consisting of 56 genes, also displayed a downregulation pattern (Fig. 2A, 2B). Genes comprised in this module showed a faster response to CIT, reaching a stable point at the T4 time point (Fig. 2A). In ME2, however, the difference between T4 and T5 is visibly larger (Fig. 2A). Within the ME4 cluster, 25% of genes were identified as apoptosis-related, while 16.1% were associated with EMT and hypoxia, respectively. Furthermore, 8.93% were classified as reference genes and 33.9% as 'other' (Fig. 2A, 2B). ORA GO analysis (FDR < 0.05) in ME4 identified pathways connected to numerous metabolic processes, cell-cell signaling and homeostasis. Similarly, KEGG pathway analysis revealed significant metabolic and cancer-related processes, including glycolysis/gluconeogenesis, HIF-1 signaling pathway, and pathways related to gastric cancer (S7 Table).

Fig. 2: Weighted Gene Co-Expression Network Analysis (WGCNA) of the expression profiles of all 54 samples using the normalized batch-corrected expression profiles of 1419 genes. A. Two WGCNA modules significantly associated with the time course phenotype (ME2 and ME4, adjusted p-value 0.037 and 0.018, respectively) The y-axis on the boxplots represents module eigengene expression levels. B. Heatmaps of gene expression of ME2 and ME4 module genes; module eigengene provide summarized representation of the expression pattern of all genes in a given module and sample type. C. Pie charts displaying the percentage of transcripts associated with particular biological processes or functions: apoptosis/cell death, hypoxia, epithelial-mesenchymal transition, reference genes, and 'other' in each module, ME2 and ME4, respectively. T0 - time point 0, frozen immediately after resection; T1 - time point 1, frozen 10 minutes after resection; T2 - time point 2, frozen 20 minutes after

resection; T3 - time point 3, frozen 30 minutes after resection; T4 - time point 4, frozen 45 minutes after resection; T5 - time point 5, frozen 60 minutes after resection.

Discussion

We examined the impact of up to 60 minutes of CIT on the transcription of cancer-related genes, with samples snap-frozen at defined time points. RNA integrity (RIN) varied across samples without a clear correlation to CIT (S1 Table), consistent with studies showing minimal RNA degradation after 3–4 hours (12,13,34,35). Despite applying four rinsing steps, colon mucosa samples showed relatively low RNA quality (mean RIN = 4.4, range 2.7 – 7.7), aligning with findings from other tissue comparisons (14,15). This may stem from enzymatic activity, bacterial influence, or tissue compartment differences (36). To mitigate this, RNA fragmentation conditions were optimized during the process of NGS library preparation.

Although utilizing a comprehensive transcriptomic approach covering over 600 genes, we detected no DEGs within the first 30 minutes post-resection, indicating this timeframe is likely safe for tissue excision and snap-freezing. Only six DEGs were identified between the 0 and 45 minute time points (T4), while 44 DEGs emerged between the 0 and 60 minute time points (T5), highlighting this period as critical for tissue freezing. Notably, five of the six DEGs in T4 and 29 of the 44 DEGs in T5 were downregulated. These results align with findings from Aktas *et al*., who reported 41 transcripts affected by prolonged CIT, including genes involved in cell cycle regulation, apoptosis, stress responses, and cancer progression (10). Although this number seems modest, it might have significant implications for researchers focusing on specific genes, gene signatures, or pathways, particularly because these DEGs are closely linked to cancer development and progression. The broader impact of sample handling on the transcriptome varies widely across studies. von der Heyde *et al*. demonstrated that cold

ischemia times exceeding 15 minutes significantly impact the expression of mRNAs, proteins, and phosphosites in tumor and normal tissues across colorectal cancer, hepatocellular carcinoma, lung adenocarcinoma, and lung squamous cell carcinoma. The authors conclude that ischemia time is a crucial quality parameter for tissue collections used for target discovery and validation in cancer research (37). Using microarrays, Musella *et al*. found minimal effects, detecting only 0.2% DEGs in normal colon tissue and colorectal carcinoma within 6 hours, though two identified DEGs were oncogenes (17). In contrast, Spruessel *et al*. applying the same genotyping technique, reported significant changes, with 20% of detectable genes and proteins altered within 30 minutes post-excision (1). Grizzle *et al.* noted that while most studies found only 1–3% of transcripts affected, they often overlooked the functional impact of these genes (35). Our findings underscore that while RNA integrity remains stable within the first 30 minutes, transcriptional changes begin to emerge beyond this point. Even within a 60-minute CIT window, the occurring transcriptional changes can potentially alter the results of studies exploring cancer and adjacent tissue genetics. These observations highlight the importance of rapid tissue processing for accurate transcriptomic analyses in cancer research.

Our analysis focused on genes involved in cancer development and progression, as they are crucial for the studies of tumor margins and tissues adjacent to the tumor, where changes in the transcriptome may be subtle (21). The results demonstrate the impact of hypoxia on EMT, a process crucial for embryonic development and tissue regeneration, which is aberrantly reactivated in cancer to drive progression and metastasis through enhanced migration, invasiveness, stemness, and resistance to therapies (38). Genes such as SNAI, CDH1, MMP9, and S100A8, which play critical roles in the EMT in cancer, exhibited significantly altered

expression levels (adjusted p-value < 0.05) in our analysis. Snail (SNAI1) and Slug (SNAI2) are transcription factors known to bind to the promoter of CDH1, encoding E-cadherin, to repress its transcription (39). The suppression of CDH1, regulated by SNAI, is a key driver of EMT, contributing to cancer progression by promoting invasion and metastasis, and is recognized as a marker of malignancy and poor clinical prognosis (40). In our study, prolonged CIT caused significant alterations in the expression of SNAI and CDH1, with upregulation of SNAI and downregulation of CDH1. Moreover, the observed upregulation of MMP9, likely induced by CIT, may reflect further malignant changes as MMP family members facilitate cancer cell invasion and metastasis (38). These changes, often linked to malignancy, may be misinterpreted as early indicators of tumor progression in research on adjacent tissues. However, our findings suggest that these alterations result from prolonged CIT.

To gain an insight into complex biological mechanisms affected by CIT, we performed enrichment analysis of DEGs using two complementary approaches: a direct analysis of all DEGs and a WGCNA-based analysis of the two resulting modules, both evaluated through GO and KEGG. The direct DEG analysis provided a broad view of the pathways enriched across all DEGs, whereas WGCNA highlighted context-specific pathways within co-regulated gene modules.

GO and KEGG analyses of all DEGs identified malignancy-associated pathways, including apoptosis, ABC transporters, and TNF signaling, as well as stress response pathways (S4 Table). In comparison, other transcriptomic studies identified alteration of pathways linked to apoptosis, stress responses, cell cycle regulation, and cancer progression (10), as well as those associated with stimulus response and signal transduction (34). Bray *et al*. found no consistent gene ontology or pathway among 168 transcripts altered within 120 minutes of

CIT, highlighting a complex cellular response (16). In our study, despite targeting over 600 cancer-related genes, pathway enrichment was broad, supporting Bray *et al*.'s conclusions (S4 Table).

WGCNA identified two significantly downregulated co-expressed gene modules, ME2 and ME4, with distinct functional patterns (S7 Table). ME2 was primarily linked to apoptosis, with GO highlighting pathways like the execution phase of apoptosis and extrinsic apoptotic signaling, while KEGG linked it to FoxO, Hippo, and p53 signaling pathways. These findings underscore CIT's significant impact on apoptosis-related pathways within 60 minutes, potentially confounding studies that do not account for its effects. ME4 was enriched for metabolic pathways, including catabolic processes, homeostasis, pyruvate metabolism (GO), glycolysis/gluconeogenesis, and choline metabolism in cancer (KEGG). Both modules featured HIF-1 and cancer-related pathways, illustrating CIT’s dual impact on apoptosis and metabolism, complicating transcriptomic interpretations. These findings align with reports of ischemia-induced changes in CRC, affecting oncogenes and histone-related genes involved in nucleosome organization, cell cycle, DNA replication, and p53 signaling, beginning at 8–10 minutes (1) and persisting up to 60 minutes (17). Notably, ME4 exhibited minimal differences between T4 and T5, whereas ME2 showed a more pronounced variation between these time points, suggesting a slower reaction to CIT (Fig. 2A).

Our study highlights that beyond RNA degradation, ischemic stress actively alters gene expression, emphasizing the need to standardize CIT in human tissue collection. The consistency of our observations suggests these transcriptomic changes stem from surgical and tissue handling procedures rather than malignancy. A key strength of our study is the use of NGS RNA-seq, which offers greater sensitivity and accuracy than microarrays (11,17),

enabling robust detection of gene expression changes, even at low levels. Unlike previous studies, our approach analyzes over 600 genes, allowing a broader transcriptomic assessment. However, limitations include a small sample size and potential influences from enzymatic activity, warm ischemia, and other preanalytical factors (2,41). Additionally, our analysis used a targeted cancer-related gene panel, which does not capture all transcriptomic changes.

Conclusion

Our study highlights the significant impact of CIT on the transcriptomic profile of healthy colon mucosa, with a particular focus on cancer-related genes. Using targeted RNA sequencing and robust analyses, we identified differentially expressed genes primarily associated with apoptosis, hypoxia, and EMT, as well as pathways involved in cancer development and progression. Our findings underscore the importance of considering CIT as a critical preanalytical variable, as prolonged ischemia can alter gene expression in ways that may mimic malignant changes, leading to misinterpretations of results and incorrect clinical conclusions. Importantly, our results suggest that CIT should be kept below 45 minutes to minimize its impact on transcriptomic profiles. However, as the effects of CIT might be tissue-dependent, this recommendation should be applied with caution when extrapolating to other tissues.

Acknowledgements

We thank all the anonymous patients for acceptance to participate, sample contribution and information provided in the questionnaire. We also thank surgeons, and nurses involved in the patient recruitment process, collaborating technicians, diagnosticians and pathologists from University Clinical Centre in Gdańsk, University Hospital in Cracow and Specialist

Hospital in Kościerzyna. This study was sponsored by the Foundation for Polish Science (FNP) under the International Research Agendas Program to J.P.D. and A.P., co-financed by the European Union under the European Regional Development Fund.

References

1. Spruessel A, Steimann G, Jung M, Lee SA, Carr T, Fentz AK, et al. Tissue ischemia time affects gene and protein expression patterns within minutes following surgical tumor excision. Biotechniques [Internet]. 2004 [cited 2024 Oct 15];36(6). Available from: https://www.tandfonline.com/action/journalInformation?journalCode=ibtn20
2. Galissier T, Schneider C, Nasri S, Kanagaratnam L, Fichel C, Coquelet C, et al. Biobanking of Fresh-Frozen Human Adenocarcinomatous and Normal Colon Tissues: Which Parameters Influence RNA Quality? PLoS One. 2016 Apr 28;11(4):e0154326.
3. Conroy MC, Lacey B, Bešević J, Omiyale W, Feng Q, Effingham M, et al. UK Biobank: a globally important resource for cancer research. Vol. 128, British Journal of Cancer. Springer Nature; 2023. p. 519–27.
4. Filipowicz N, Drężek K, Horbacz M, Wojdak A, Szymanowski J, Rychlicka-Buniowska E, et al. Comprehensive cancer-oriented biobanking resource of human samples for studies of post-zygotic genetic variation involved in cancer predisposition. PLoS One. 2022 Apr 1;17(4 April).
5. Bonizzi G, Zattoni L, Capra M, Cassi C, Taliento G, Ivanova M, et al. Standard operating procedures for biobank in oncology. Front Mol Biosci. 2022 Aug 26;9.
6. Foley ME, Vinson AJ, Skinner TAA, Kiberd BA, Tennankore KK. The Impact of Combined Warm and Cold Ischemia Time on Post-transplant Outcomes. Can J Kidney Health Dis. 2023 Jan 1;10.
7. Pedersen IS, Thomassen M, Tan Q, Kruse T, Thorlacius-Ussing O, Garne JP, et al. Differential effect of surgical manipulation on gene expression in normal breast tissue and breast tumor tissue. Molecular Medicine. 2018 Dec 16;24(1):57.
8. Ma Y, Dai H, Kong X. Impact of warm ischemia on gene expression analysis in surgically removed biosamples. Anal Biochem. 2012 Apr;423(2):229–35.
9. Shabihkhani M, Lucey GM, Wei B, Mareninov S, Lou JJ, Vinters H V., et al. The procurement, storage, and quality assurance of frozen blood and tissue biospecimens in pathology, biorepository, and biobank settings. Clin Biochem. 2014 Mar;47(4–5):258–66.
10. Aktas B, Sun H, Yao H, Shi W, Hubbard R, Zhang Y, et al. Global gene expression changes induced by prolonged cold ischemic stress and preservation method of breast cancer tissue. Mol Oncol. 2014 May 17;8(3):717–27.
11. Liu NW, Sanford T, Srinivasan R, Liu JL, Khurana K, Aprelikova O, et al. Impact of Ischemia and Procurement Conditions on Gene Expression in Renal Cell Carcinoma. Clinical Cancer Research. 2013 Jan 1;19(1):42–9.
12. Bao WG, Zhang X, Zhang JG, Zhou WJ, Bi TN, Wang JC, et al. Biobanking of Fresh-frozen Human Colon Tissues: Impact of Tissue Ex-vivo Ischemia Times and Storage Periods on RNA Quality. Ann Surg Oncol. 2013 May 19;20(5):1737–44.
13. Micke P, Ohshima M, Tahmasebpoor S, Ren ZP, Östman A, Pontén F, et al. Biobanking of fresh frozen tissue: RNA is stable in nonfixed surgical specimens. Laboratory Investigation. 2006 Feb;86(2):202–11.

14. Viana CR, Neto CS, Kerr LM, Palmero EI, Marques MMC, Colaiacovo T, et al. The interference of cold ischemia time in the quality of total RNA from frozen tumor samples. Cell Tissue Bank. 2013 Jun;14(2):167–73.
15. Zeugner S, Mayr T, Zietz C, Aust DE, Baretton GB. RNA quality in fresh-frozen gastrointestinal tumor Specimens—Experiences from the tumor and healthy tissue bank TU dresden. Recent Results in Cancer Research. 2015;199:85–93.
16. Bray SE, Paulin FEM, Fong SC, Baker L, Carey FA, Levison DA, et al. Gene expression in colorectal neoplasia: Modifications induced by tissue ischaemic time and tissue handling protocol. Histopathology. 2010 Jan;56(2):240–50.
17. Musella V, Verderio P, Reid JF, Pizzamiglio S, Gariboldi M, Callari M, et al. Effects of Warm Ischemic Time on Gene Expression Profiling in Colorectal Cancer Tissues and Normal Mucosa. PLoS One. 2013 Jan 7;8(1):e53406.
18. Zhou L, Xu G, Huang F, Chen W, Zhang J, Tang Y. Apoptosis related genes mediated molecular subtypes depict the hallmarks of the tumor microenvironment and guide immunotherapy in bladder cancer. BMC Med Genomics. 2023 Dec 1;16(1).
19. Li D, Li L, Yang W, Chen L, Chen X, Wang Q, et al. Prognostic values of SNAI family members in breast cancer patients. Ann Transl Med. 2020 Aug;8(15):922–922.
20. Van 't Veer LJ, Bernards R. Enabling personalized cancer medicine through analysis of gene-expression patterns. Vol. 452, Nature. Nature Publishing Group; 2008. p. 564–70.
21. Andreou M, Jąkalski M, Duzowska K, Filipowicz N, Kostecka A, Davies H, et al. Prelude to malignancy: A gene expression signature in normal mammary gland from breast cancer patients suggests pre-tumorous alterations and is associated with adverse outcomes. Int J Cancer. 2024 Nov 8;155(9):1616–28.
22. Mieczkowska A, Schumacher A, Filipowicz N, Wardowska A, Zieliński M, Madanecki P, et al. Immunophenotyping and transcriptional profiling of in vitro cultured human adipose tissue derived stem cells. Sci Rep. 2018 Jul 27;8(1):11339.
23. Matsubara T, Soh J, Morita M, Uwabo T, Tomida S, Fujiwara T, et al. DV200 Index for Assessing RNA Integrity in Next-Generation Sequencing. Biomed Res Int. 2020 Jan 27;2020(1).
24. Andreou M, Jąkalski M, Duzowska K, Filipowicz N, Kostecka A, Davies H, et al. Prelude to malignancy: A gene expression signature in normal mammary gland from breast cancer patients suggests pre-tumorous alterations and is associated with adverse outcomes. Int J Cancer. 2024 Nov 8;155(9):1616–28.
25. Dobin A, Davis CA, Schlesinger F, Drenkow J, Zaleski C, Jha S, et al. STAR: ultrafast universal RNA-seq aligner. Bioinformatics. 2013 Jan 1;29(1):15–21.
26. Robinson MD, Oshlack A. A scaling normalization method for differential expression analysis of RNA-seq data. Genome Biol. 2010 Mar 2;11(3):R25.
27. Lê S, Josse J, Husson F. **FactoMineR** : An *R* Package for Multivariate Analysis. J Stat Softw. 2008;25(1).
28. Zhang Y, Parmigiani G, Johnson WE. ComBat-seq: batch effect adjustment for RNA-seq count data. NAR Genom Bioinform. 2020 Sep 1;2(3).
29. Langfelder P, Horvath S. WGCNA: an R package for weighted correlation network analysis. BMC Bioinformatics. 2008 Dec 29;9(1):559.
30. Ritchie ME, Phipson B, Wu D, Hu Y, Law CW, Shi W, et al. limma powers differential expression analyses for RNA-sequencing and microarray studies. Nucleic Acids Res. 2015 Apr 20;43(7):e47–e47.

31. Liao Y, Wang J, Jaehnig EJ, Shi Z, Zhang B. WebGestalt 2019: gene set analysis toolkit with revamped UIs and APIs. Nucleic Acids Res. 2019 Jul 2;47(W1):W199–205.
32. Matsubara T, Soh J, Morita M, Uwabo T, Tomida S, Fujiwara T, et al. DV200 Index for Assessing RNA Integrity in Next-Generation Sequencing. Biomed Res Int. 2020 Jan 27;2020(1).
33. Vasaikar S V., Deshmukh AP, den Hollander P, Addanki S, Kuburich NA, Kudaravalli S, et al. EMTome: a resource for pan-cancer analysis of epithelial-mesenchymal transition genes and signatures. Br J Cancer. 2021 Jan 5;124(1):259–69.
34. Borgan E, Navon R, Vollan HKM, Schlichting E, Sauer T, Yakhini Z, et al. Ischemia caused by time to freezing induces systematic microRNA and mRNA responses in cancer tissue. Mol Oncol. 2011 Dec 29;5(6):564–76.
35. Grizzle WE, Otali D, Sexton KC, Atherton DS. Effects of Cold Ischemia on Gene Expression: A Review and Commentary. Biopreserv Biobank. 2016 Dec;14(6):548–58.
36. Heumüller-Klug S. Degradation of intestinal mRNA: A matter of treatment. World J Gastroenterol. 2015;21(12):3499.
37. von der Heyde S, Raman N, Gabelia N, Matias-Guiu X, Yoshino T, Tsukada Y, et al. Tumor specimen cold ischemia time impacts molecular cancer drug target discovery. Cell Death Dis. 2024 Sep 26;15(9):691.
38. Huang Y, Hong W, Wei X. The molecular mechanisms and therapeutic strategies of EMT in tumor progression and metastasis. Vol. 15, Journal of Hematology and Oncology. BioMed Central Ltd; 2022.
39. Tam SY, Wu VWC, Law HKW. Hypoxia-Induced Epithelial-Mesenchymal Transition in Cancers: HIF-1α and Beyond. Front Oncol. 2020 Apr 8;10.
40. Cano A, Pérez-Moreno MA, Rodrigo I, Locascio A, Blanco MJ, del Barrio MG, et al. The transcription factor Snail controls epithelial–mesenchymal transitions by repressing E-cadherin expression. Nat Cell Biol. 2000 Feb 13;2(2):76–83.
41. Medeiros F, Rigl CT, Anderson GG, Becker SH, Halling KC. Tissue Handling for Genome-Wide Expression Analysis: A Review of the Issues, Evidence, and Opportunities. Arch Pathol Lab Med. 2007 Dec 1;131(12):1805–16.

Supporting information

S1 Table. Pseudonymized list of patients that were included in the study with the information on gender, ICD10 (International Statistical Classification of Diseases and Related Health Problems, 10th Revision), sample time points, type of tissue, and RNA Integrity Number (RIN) values. (XLSX)

S2 Table. Target genes included in the designed panel and functional classification of particular transcripts. (a) Gene symbols of targets included in the panel, (b) Ensembl ID, (c)

Ensembl ID as in Gencode (version 35), (d) Gene symbol according to the HUGO Gene Nomenclature Committee, (e) EntrezID, (f) Full name of the gene, (g) - (p) Functional classification. **(XLSX)**

S3 Table. Differentially Expressed Genes (DEGs) between time points T5 (60 minutes) and T0 (0 minutes). (a) Ensembl ID as in Gencode (version 35), (b) Gene symbol, (c) Direction of the expression change: up - upregulation, down - downregulation, (d) logFoldChange (logFC), (e) p-value, (f) False Discovery Rate (FDR), (g) Indication if the gene was included in our panel: N - no, Y - yes, (h) Information regarding the comparison between different groups; T5 (time point 5, 60 minutes after resection), T4 (time point 4, 45 minutes after resection) and T0 (time point 0, 0 minutes after resection). **(XLSX)**

S4 Table. The results of enrichment analysis for differentially expressed genes (DEGs) between T5 (time point 5, 60 minutes after resection) and T0 (time point 0, 0 minutes after resection). (a) Gene Ontology Term ID and Kyoto Encyclopedia of Genes and Genomes (KEGG) ID, (b) short description of identified Gene Ontology (GO) terms, (c) p-value, (d) False Discovery Rate (FDR), (e) ratio of genes from our targeted panel vs. all genes associated with the particular GO term, (f) genes from our analysis associated with the particular GO term, (g) information regarding the comparison between different groups; T5 (time point 5, 60 minutes after resection) and T0 (time point 0, 0 minutes after resection). **(XLSX)**

S5 Table. Genes included in weighted gene co-expression network analysis (WCGNA) module eigengene 2 (ME2). (a) gene symbol, (b) description, (c) functional classification. **(XLSX)**

S6 Table. Genes included in weighted gene co-expression network analysis (WCGNA) module eigengene 4 (ME4). (a) gene symbol, (b) description, (c) functional classification. **(XLSX)**

S7 Table. Gene Ontology (GO) terms and KEGG pathways identified via enrichment analyses, including cluster membership information. Enrichment analysis was conducted for genes from weighted gene co-expression network analysis (WCGNA) from statistically significant modules - ME2 and ME4. (a) Gene Ontology Term ID and Kyoto Encyclopedia of Genes and Genomes (KEGG) ID, (b) short description of identified Gene Ontology (GO) terms,

(c) p-value, (d) False Discovery Rate (FDR), (e) ratio of genes included in the panel, associated with the particular GO term, (f) Genes associated with the particular GO term, (g) Information regarding the comparison between different groups: T5 (time point 5, 60 minutes after resection) and T0 (time point 0, 0 minutes after resection). **(XLSX)**

Figure 1

Click here to access/download;Figure;Fig1.tif

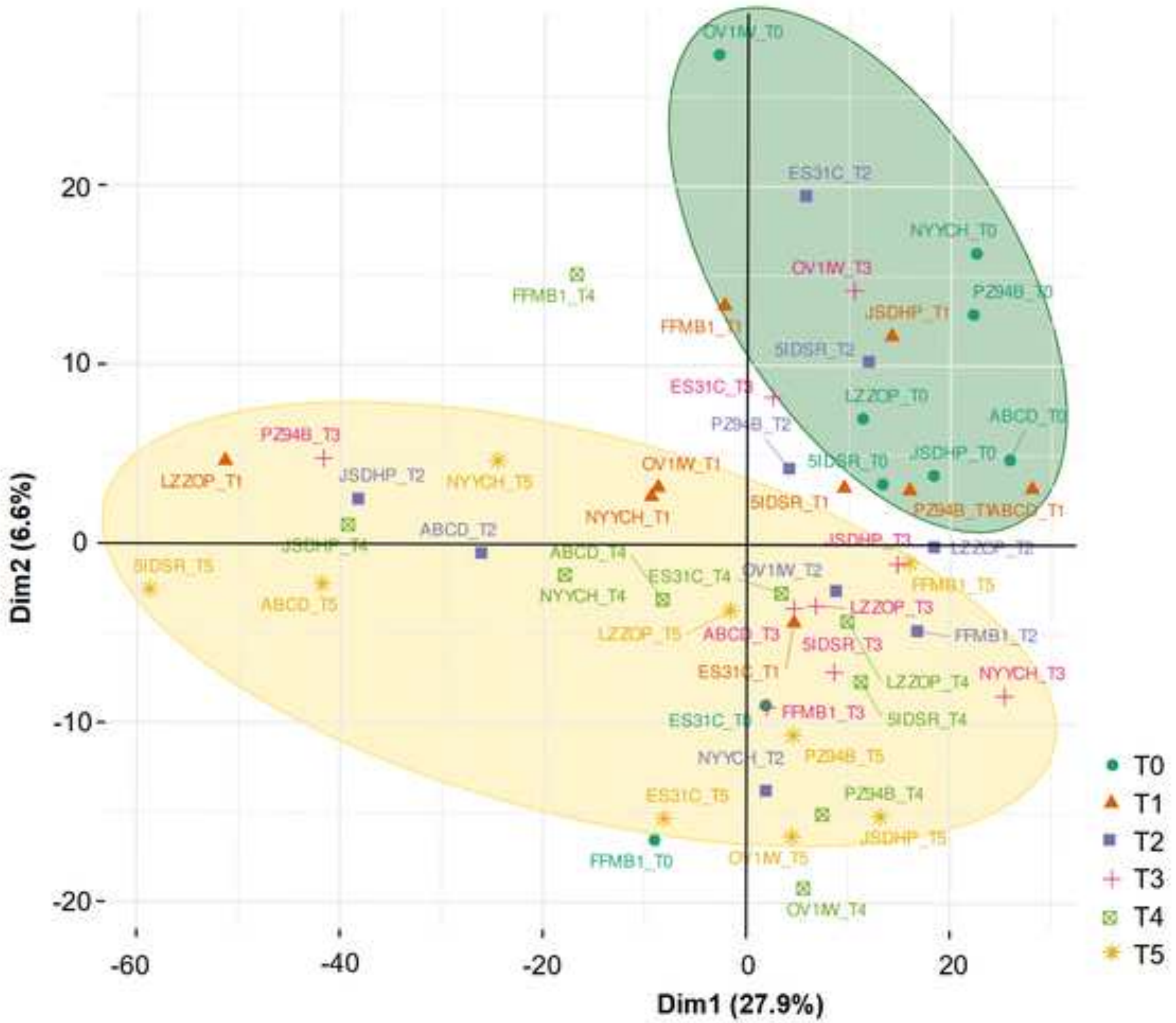

Figure 2

Click here to access/download;Figure;Fig2.tif

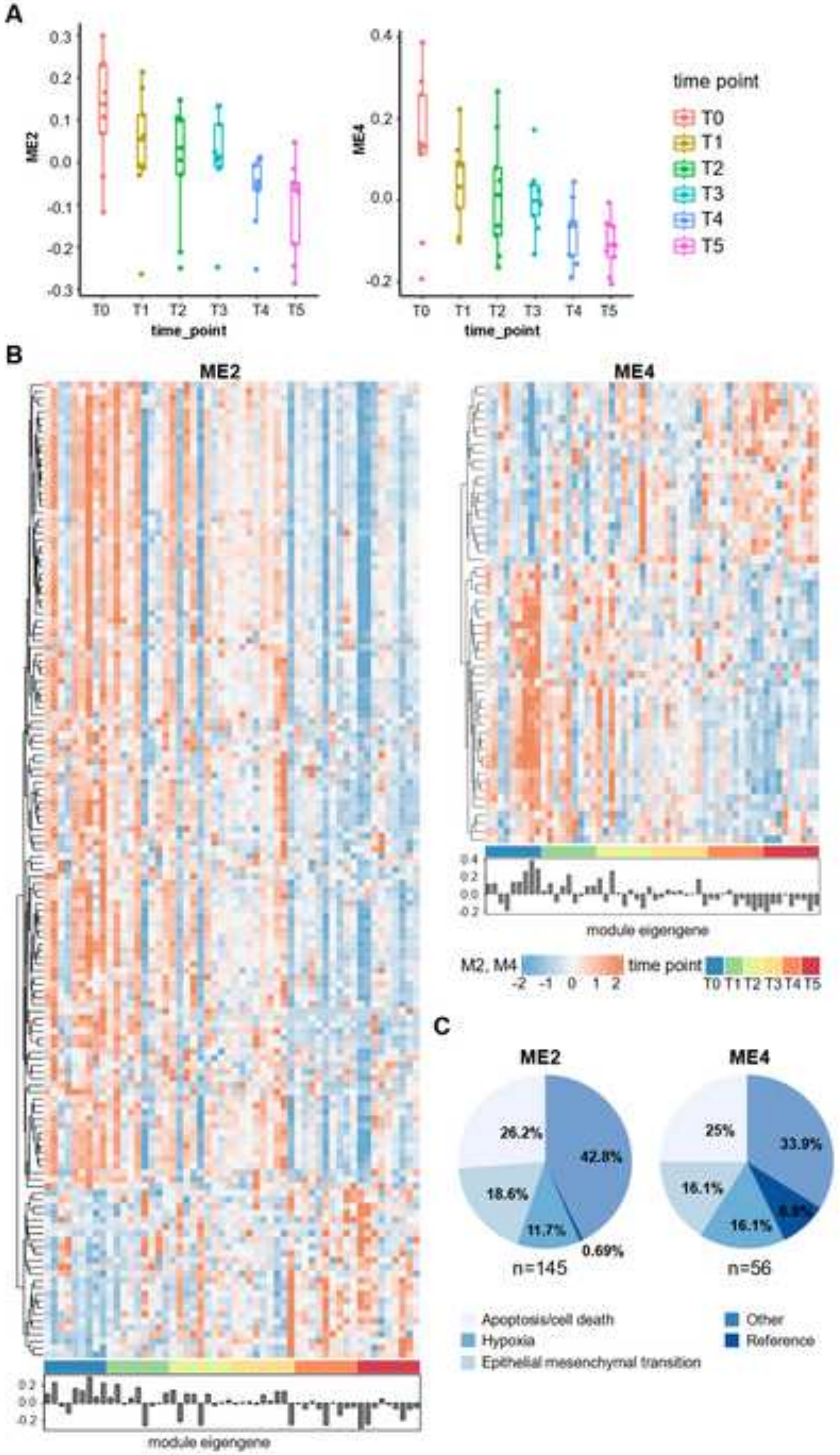

Click here to access/download
Supporting Information
Duzowska_et_al_Supplementary_tables.xlsx

The effect of cold ischemia time on hypoxia, EMT, and apoptosis pathways in normal colon mucosa

Katarzyna Duzowska[1], Mikołaj Opiełka[1,2], Kinga Drężek-Chyła[1], Anna Kostecka[1], Monika Horbacz[1], Jarosław Skokowski[3], Olga Rostkowska[4], Jarosław Kobiela[4], Leszek Kalinowski[5,6], Jan P. Dumanski[1,7], Arkadiusz Piotrowski[1], Marcin Jąkalski[1,8&]*, Natalia Filipowicz[1&]*

[1] 3P-Medicine Laboratory, Medical University of Gdańsk, Gdańsk, Poland

[2] Department of Biochemistry, Medical University of Gdańsk, Gdańsk, Poland

[3] Academy of Applied Medical and Social Science, Elbląg, Poland

[4] Department of Oncological, Transplant and General Surgery, Medical University of Gdańsk, Gdańsk, Poland

[5] Department of Medical Laboratory Diagnostics, Medical University of Gdańsk, Gdańsk, Poland

[6] BioTechMed Center, Department of Mechanics of Materials and Structures, Gdańsk University of Technology, Gdańsk, Poland

[7] Department of Immunology, Genetics and Pathology and Science for Life Laboratory, Uppsala University, Uppsala, Sweden

[8] Center for Applied Genomics and Bioinformatics, Faculty of Biology, University of Gdańsk, Gdańsk, Poland

[&]these authors contributed equally to this work

* Corresponding author:

Email: natalia.filipowicz@gumed.edu.pl and marcin.jakalski@ug.edu.pl

Short title

Cold ischemia time influences transcriptional landscape of colon mucosa

Keywords

cold ischemia, hypoxia, post-zygotic mutations, preanalytical variable, epithelial-to-mesenchymal transition (EMT), targeted RNA sequencing, colon mucosa, non-tumorous tissue, differentially expressed genes, weighted gene co-expression network analysis (WGCNA)

Abstract

Cold ischemia time (CIT), the interval between tissue excision and preservation, is a critical preanalytical variable that profoundly impacts gene expression profiles. Variability in CIT can lead to inconsistent transcriptomic results, making study interpretation challenging and undermining reproducibility in biomedical research. Our study aimed to evaluate the impact of CIT on the expression of cancer-related genes, particularly these involved in hypoxia, apoptosis, and epithelial-to-mesenchymal transition (EMT). We performed RNA sequencing on 54 normal colon mucosa samples from nine patients undergoing colorectal cancer surgeries, freezing samples at predefined intervals ranging from 0 to 60 minutes. A total of 44 differentially expressed genes (DEGs) ($p < 0.05$) were identified when comparing samples frozen immediately (T0) with those frozen after 60 minutes (T5). These DEGs were further analyzed through functional and pathway enrichment analyses and weighted gene co-expression network analysis (WGCNA). The enrichment analysis revealed significant alterations in pathways associated with apoptosis, hypoxia, EMT, and cancer progression,

including p53 and HIF-1 signaling. WGCNA highlighted two co-expressed gene modules: ME2, which showed downregulation of apoptosis-related genes, and ME4, linked to apoptosis and cellular metabolism. Our findings highlight CIT as a critical preanalytical variable, showing that prolonged ischemia can induce transcriptomic changes that may mimic malignancy, and potentially confound research outcomes. To minimize such effects, we recommend keeping CIT under 45–60 minutes.

Introduction

The quality and integrity of biological samples are crucial for multi-omic studies, directly impacting the reliability of results. Preanalytical variables, including patient-specific factors and tissue handling protocols, can significantly impact the outcomes (1,2). With biobanking playing a crucial role in biomedical translational research, standardized tissue collection protocols are essential to minimize external variability (3–5). Ischemia, defined as a restriction of blood supply to tissues or organs, results in a shortage of oxygen supply necessary to sustain the cellular metabolism. In the *ex vivo* context, this process can be classified into warm and cold ischemia. Cold ischemia time (CIT) refers to the condition in which oxygen supply is entirely absent after tissue excision while the sample is maintained at temperatures below body temperature (6). Warm ischemia time (WIT) refers to the period during which the tissue remains in the donor's body but the oxygen supply is insufficient to meet metabolic demands. This preanalytical variable is challenging to control as it relies on the efficiency of the surgical team, for which the priority is the successful completion of the operation. Reports on the impact of WIT on transcriptomic profiles are scarce, but studies, including those by Pedersen *et al*. and Ma *et al*., highlight its effects, identifying numerous differentially expressed genes (DEGs) and alterations in oncogenic, inflammatory, and immunological

pathways (7,8). In contrast, CIT is determined by the biobanking procedure and can be effectively managed by the tissue collector (9). Prolonged CIT, rather than malignancy, can lead to specific, even subtle, changes in gene expression profiles, complicating the interpretation of tissue samples.

Several studies have assessed the impact of CIT on RNA quality, measured as RNA integrity number (RIN), reporting minimal changes up to 4 hours (10–12) and even up to 16 hours at room temperature (13). RNA quality also varies by tissue type, with thyroid and colorectal tissues being more sensitive to cold ischemia compared to stomach and lung tissues (14). Gastrointestinal samples, in general, tend to exhibit lower RNA quality than those from other organs (2,15), with tumor tissues displaying significantly higher RINs than normal tissues, though CIT has little effect on this observed difference (2).

While most reports focus on RNA integrity, few have explored the impact of CIT on gene expression, particularly in cancer-related pathways (11,16,17). Transcriptomic analyses suggest that CIT exceeding 60 minutes can significantly alter gene expression. Aktas *et al*. identified 41 transcripts affected by prolonged CIT, including apoptosis- and cell cycle-related genes, with over 3% showing significant changes (10). Other study reported transcriptomic alterations as early as 30 minutes post-excision, for instance delayed freezing of colorectal carcinoma samples lead to increased KLF6 expression, potentially misrepresenting its role in tumor pathogenesis (16). A study of transcriptomic profiles in renal carcinoma showed over 4,000 genes were affected at longer CIT durations and higher temperatures, emphasizing the need for immediate snap-freezing to preserve gene expression profiles relevant to cancer research (11).

Despite these findings, the existing literature remains limited, particularly regarding the effect of cold ischemia time (CIT) on the transcription of cancer-associated genes, including those involved in epithelial-to-mesenchymal transition (EMT), hypoxia, apoptosis, and cell death. These genes are crucial for understanding cancer biology and the complex interactions within the tumor microenvironment (18). Moreover, their expression can directly influence treatment decisions and patient outcomes (16,18–20). Some of these genes may be first responders to cold ischemia, making them potential markers of CIT. Examining these preanalytical factors is essential not only to enhance the reliability and reproducibility of experimental findings but also to ensure that scientific conclusions translate effectively into clinical practice.

The primary aim of this research is to investigate the influence of CIT on the transcription profile of macro- and microscopically normal colon mucosa collected from patients with colorectal cancer. The focus on normal colon mucosa aligns with the design of ongoing research in our laboratory, which investigates genetic mosaicism focusing on post-zygotic mutations accumulating in morphologically normal tissues proximal and distal to tumors (4,21). Furthermore, the majority of the publicly available studies focused on tumor tissue, with limited insights into healthy tissue (2,13,17). Here we employed targeted RNA sequencing (NGS RNAseq), including, among the others, genes associated with hypoxia, apoptosis, and cancer, thus addressing a critical gap in the literature on preanalytical variables for tissue collection and subsequent analysis for cancer research.

Materials and methods

Study Design and Sample Handling

The healthy colon mucosa samples were collected from 9 consecutive patients (2 females; 7 males; mean age 68 years ± 10.9) diagnosed with colorectal cancer who underwent surgical resection 21st of April to 24th of August 2021 in the Department of Oncological, Transplant, and General Surgery; University Clinical Center in Gdansk. The summary of patients' data is presented in Table 1. After the surgical resection, a larger, full-walled specimen of the colon was immediately excised approximately 10 - 15 cm away from the primary tumor, rinsed in saline and left at room temperature until the mucosa collection procedure at a particular time point was completed. At each time point, a sample of macroscopically unaffected mucosa was excised by dissecting from the colon specimen and detaching gently from the muscular layer. Subsequent washing steps were implemented, including: saline solution (twice), antibiotic solution (Penicillin - Streptomycin 5000 U/ml), and saline solution, followed by snap freezing in liquid nitrogen. Tissue fragments were collected at six time points - 0 (T0), 10 (T1), 20 (T2), 30 (T3), 45 (T4), and 60 (T5) minutes after the surgical resection, estimated as the time of cold ischemia. Sample collected at T0 was processed immediately after organ resection. First time point (T0) was used as the reference and the starting point for all the subsequent analyses. In total 54 samples were collected and subjected to further analysis.

RNA Extraction and Quality Control

Colon mucosa samples were stored at -80°C until RNA isolation. Total RNA was extracted from 10 - 30 mg of tissue samples that were mechanically homogenized using T10 Basic ULTRA - TURRAX disperser (IKA) in the presence of QIAzol Lysis reagent (Qiagen). RNA isolation, purification, and DNase digestion were performed using the RNeasy Mini kit (Qiagen) according to the original protocol with two modifications as described in the previous paper (22) RNA quality and quantity were assessed using TapeStation (Agilent Technologies) using

RNA ScreenTape kit according to the manufacturer's protocol (Table 2). RNA integrity number (RIN) was calculated using TapeStation analysis software (Agilent Technologies). Additionally, the DV200 index, indicating the percentage of RNA fragments > 200 nt, was calculated and used as a quality assessment standard (23). Samples with a DV200 index > 75% were used for further NGS analysis.

Targeted RNA sequencing

The targeted RNA sequencing panel was designed with the Roche NimbleDesign online tool (Roche, now HyperDesign, https://hyperdesign.com/#/) and covered 7229 regions with a total length of 1,243,523 bp. It included 634 genes based on literature research and covered genes associated with apoptosis, cell death, hypoxia, epithelial-to-mesenchymal transition, and other cancer-related genes (the full list of transcripts is given in S2 Table). NGS libraries were prepared with the Kapa RNA HyperPrep Kit (Roche) using Automated Liquid Handling Bravo NGS workstation (Agilent Technologies) according to the manufacturer manual with 100 ng RNA used as an input and enzymatic fragmentation at 94°C for 6 minutes, with the addition of ERCC RNA Spike-In Mix (Invitrogen) as an external RNA control. All the single libraries were multiplexed and hybridized with SeqCap EZ Choice Probes (Roche) designed by our group and KAPA HyperCapture Reagent and Bead kit (v2, Roche Sequencing Solutions, Inc.) according to SeqCap RNA Enrichment System User's Guide (v.1.0) with slight modifications. Component A was replaced with formaldehyde, and the Multiplex Hybridization Enhancing Oligo Pool was replaced with Universal Blocking Oligos (UBO).The hybridization was run for 18h, the library was cleaned up, post-captured, amplified, and purified, followed by inspection of fragment distribution using TapeStation with High Sensitivity D1000 Screen Tape kit (Agilent Technologies) and qPCR quantitation in a Roche

LightCycler 480 with KAPA Library Quantification Kit (Roche). Paired-end reads of 150 bp were generated using TruSeq RNA Access sequencing chemistry by an external service provider (Macrogen Europe, Amsterdam, The Netherlands).

Transcriptomic data analysis

The RNA-seq data were processed as described in Andreou et al. 2024 (24). Briefly, after the quality filtering and trimming of the raw FASTQ files with BBDuk (https://sourceforge.net/projects/bbmap/, version 38.36), the resulting reads were mapped to the reference human genome (hg38, GENCODE version 39) using STAR version 2.7.3a (25). Raw read counts assigned to the annotated genes obtained in the above process were collated into a single gene expression matrix and processed further in R programming language (https://www.r-project.org, version 4.1.2). Lowly expressed genes were filtered out based on a minimal required CPM expression threshold. The filtered gene expression matrix was normalized using the TMM method in edgeR (26). Principal Component Analysis (PCA) was performed to investigate sample grouping and identify potential outliers using FactoMineR, version 2.4 (27). We accounted for the paired nature of the RNAseq data by treating the patient ID as batch and removing its effect from the data using ComBat-seq ~~Batch effect correction was performed using ComBat-seq~~ (28). Significantly differentially expressed genes (DEGs) were identified with EdgeR using the glmLRT function (likelihood ratio test) with a significance threshold set to 0.05 (False Discovery Rate, FDR).

Weighted gene co-expression network analysis (WGCNA) was performed on the normalized and batch-adjusted gene expression values using the WGCNA R library WGCNA (29). Soft threshold value for building the correlation network was selected empirically based on diagnostic plots (pickSoftThreshold function). The final network was built using the

Formatted: English (United States)

blockwiseModules function, where the TOMType parameter was set to “signed”. Subsequently, a linear model (lmFit, from the limma package, (30)) was run on all modules to identify those associated with the tested trait (time point).

Overrepresented terms / GO / pathways

Overrepresentation analysis (ORA) was used to perform functional enrichment analysis of significantly differentially expressed genes, utilizing the Gene Ontology (GO) database and the genome reference set in WebGestalt (31). Pathway enrichment analysis was similarly conducted with ORA, focusing on KEGG pathways and the genome reference set in WebGestalt. In addition, functional and pathway enrichment analyses were separately applied to genes from WGCNA modules ME2 and ME4, using WebGestalt for the analysis.

Bioethics Committee Approval

All procedures for sample collection were approved by the Independent Bioethics Committee for Research at the Medical University of Gdansk (approval number NKBBN/564/2018 with multiple amendments). Written informed consent was obtained from all the patients prior to surgery. All procedures were performed in accordance with the relevant national and international laws and guidelines as well as in compliance with European Union General Data Protection Regulation (EU GDPR).

Table 1. A summary of donors and diagnoses included in the study

Patient ID	***Sex***	***Age***	***ICD-10 Classification***
ES31C	Male	67	C18.7 - Malignant neoplasm of sigmoid colon
5IDSR	Female	70	C19 - Malignant neoplasm of rectosigmoid junction
OV1IW	Male	67	C20 - Malignant neoplasm of rectum

NYYCH	Male	48	C20 - Malignant neoplasm of rectum
LZZOP	Female	72	C19 - Malignant neoplasm of rectosigmoid junction
JSDHP	Male	68	C19 - Malignant neoplasm of rectosigmoid junction
PZ94B	Male	83	C18.7 - Malignant neoplasm of sigmoid colon
FFMB1	Male	59	C20 - Malignant neoplasm of rectum
ABCD	Male	83	C18.5 - Malignant neoplasm of splenic flexure

Results

Quality of total RNA within the studied time frame

Tissues from nine patients were collected and snap-frozen at six time points: immediately post-resection (T0), 10 minutes (T1), 20 minutes (T2), 30 minutes (T3), 45 minutes (T4), and 60 minutes (T5) post-resection (see Materials and Methods). As many prior studies have focused on RNA quality as a consequence of CIT, we performed quality control by measuring both RIN and DV200 values. Notably, RIN values remained consistent across all analyzed time points, with a median value of 4.3. Furthermore, all samples had DV200 above 75%, indicating that RNA was of sufficient quality for subsequent analysis (32). The consistency of these results across all time points suggest that RNA integrity was preserved throughout the collection and processing protocol. Detailed RIN measurements for each sample are provided in S1 Table.

Differential Expression Analysis highlights genes associated with hypoxia, EMT, and apoptosis

Differential expression analysis revealed 44 significantly differentially expressed genes (DEGs) between the two extreme time points set for our study, T0 and T5 (60 min) (S3 Table). Principal component analysis (PCA) demonstrated a noticeable separation between samples at T0 and T5 after eliminating patient batch effect, as illustrated in Fig. 1. Comparisons involving T0 and other time points (T1–T4) showed fewer statistically significant DEGs: zero between T0 vs T3, and six for T0 vs T4, namely ABCB1, ABCG2, EPAS1, FZD2, LAMA3, and TMPRSS2. Our results show that the initial gene expression changes occur between 30 minutes (T3) and 45 minutes (T4) post-excision; with 44 genes displaying significant changes between 45 minutes and 60 minutes (T5) after excision (S3 Table). Thus, our further analyses focused on the T0 versus T5 comparison.

Applying an absolute logFC cutoff of ≥1, we identified 15 upregulated genes and 29 downregulated genes in T5 compared to T0. Notably, DEGs included hypoxia- and EMT-associated genes such as SNAI, CDH1, ADM, MMP9, EGLN3, IL-6, AKT3, TSPAN1, and DSC2, as well as apoptosis-related genes: CASP7, TNFSF10, DEFB1, AKT3, and JPH3. Among the 44 DEGs identified between T0 and T5 (S3 Table), 5 were linked to hypoxia, 8 to apoptosis or cell death, and 10 to epithelial-to-mesenchymal transition (EMT) determined using EMTome (33). The remaining 19 were classified as 'other'. It is important to stress that none of 22 housekeeping genes used as a reference set included in the panel showed significant expression changes. S3 Table provides the complete DEG list with the logFC, p-values, and FDR values.

Functional enrichment of DEGs using overrepresentation analysis (ORA) identified enrichment of Gene Ontology (GO) terms associated with processes such as apoptosis, cell-cell adhesion, tissue migration, and others. TNF signaling, ABC transporters, gastric cancer, and cancer pathways were among the enriched pathways shown by ORA KEGG analysis (S4 Table).

Fig. 1: Principal component analysis (PCA) of all 54 samples using the normalized batch-corrected expression profiles of 1419 genes (both targeted and non-targeted). Each point represents the orientation of a sample projected into the transcriptional space, color and symbol refer to its group membership. The analysis demonstrated clearly separated clusters of T0 and T5 samples (marked in green and yellow circles), a pattern not pronounced among the intermediate time points (T1-T4). T0 - time point 0, frozen immediately after resection; T1 - time point 1, frozen 10 minutes after resection; T2 - time point 2, frozen 20 minutes after resection; T3 - time point 3, frozen 30 minutes after resection; T4 - time point 4, frozen 45 minutes after resection; T5 - time point 5, frozen 60 minutes after resection.

Weighted Gene Co-Expression Network Analysis (WGCNA) confirms two gene modules associated with apoptosis, EMT and hypoxia

To find the clusters of highly correlated genes in our dataset, we applied the WGCNA analysis that identified two significant gene modules, ME2 and ME4 (adjusted p-values 0.037 and 0.018, respectively), which demonstrate distinct patterns during the time course of our study. Notably, the genes within these modules were associated with key biological processes, including apoptosis, EMT, and hypoxia-related pathways (Fig. 2).

ME2 consisted of 145 genes, primarily exhibiting a downregulation pattern in T5 in relation to T0 (Fig. 2A, 2B). Among these, 26.2% were related to apoptosis or cell death, 18.6% to EMT, 11.7% to hypoxia, and 0.69% (one gene) was annotated as a reference gene in our targeted panel (Fig. 2C). The remaining 42.8% were categorized as 'other' (see details in S5 Table). Functional enrichment analysis of ME2 genes using ORA GO (FDR < 0.05) identified several pathways, including those related to the execution phase of apoptosis and the extrinsic apoptotic signaling pathway. KEGG pathway analysis further highlighted nine pathways,

including cancer-related and apoptosis-related processes such as p53 signaling, HIF-1 signaling, and FoxO signaling (S7 Table).

ME4, a smaller gene module consisting of 56 genes, also displayed a downregulation pattern (Fig. 2A, 2B). Genes comprised in this module showed a faster response to CIT, reaching a stable point at the T4 time point (Fig. 2A). In ME2, however, the difference between T4 and T5 is visibly larger (Fig. 2A). Within the ME4 cluster, 25% of genes were identified as apoptosis-related, while 16.1% were associated with EMT and hypoxia, respectively. Furthermore, 8.93% were classified as reference genes and 33.9% as 'other' (Fig. 2A, 2B). ORA GO analysis (FDR < 0.05) in ME4 identified pathways connected to numerous metabolic processes, cell-cell signaling and homeostasis. Similarly, KEGG pathway analysis revealed significant metabolic and cancer-related processes, including glycolysis/gluconeogenesis, HIF-1 signaling pathway, and pathways related to gastric cancer (S7 Table).

Fig. 2: Weighted Gene Co-Expression Network Analysis (WGCNA) of the expression profiles of all 54 samples using the normalized batch-corrected expression profiles of 1419 genes. A. Two WGCNA modules significantly associated with the time course phenotype (ME2 and ME4, adjusted p-value 0.037 and 0.018, respectively) The y-axis on the boxplots represents module eigengene expression levels. B. Heatmaps of gene expression of ME2 and ME4 module genes; module eigengene provide summarized representation of the expression pattern of all genes in a given module and sample type. C. Pie charts displaying the percentage of transcripts associated with particular biological processes or functions: apoptosis/cell death, hypoxia, epithelial-mesenchymal transition, reference genes, and 'other' in each module, ME2 and ME4, respectively. T0 - time point 0, frozen immediately after resection; T1 - time point 1, frozen 10 minutes after resection; T2 - time point 2, frozen 20 minutes after

resection; T3 - time point 3, frozen 30 minutes after resection; T4 - time point 4, frozen 45 minutes after resection; T5 - time point 5, frozen 60 minutes after resection.

Discussion

We examined the impact of up to 60 minutes of CIT on the transcription of cancer-related genes, with samples snap-frozen at defined time points. RNA integrity (RIN) varied across samples without a clear correlation to CIT (S1 Table), consistent with studies showing minimal RNA degradation after 3–4 hours (12,13,34,35). Despite applying four rinsing steps, colon mucosa samples showed relatively low RNA quality (mean RIN = 4.4, range 2.7 – 7.7), aligning with findings from other tissue comparisons (14,15). This may stem from enzymatic activity, bacterial influence, or tissue compartment differences (36). To mitigate this, RNA fragmentation conditions were optimized during the process of NGS library preparation.

Although utilizing a comprehensive transcriptomic approach covering over 600 genes, we detected no DEGs within the first 30 minutes post-resection, indicating this timeframe is likely safe for tissue excision and snap-freezing. Only six DEGs were identified between the 0 and 45 minute time points (T4), while 44 DEGs emerged between the 0 and 60 minute time points (T5), highlighting this period as critical for tissue freezing. Notably, five of the six DEGs in T4 and 29 of the 44 DEGs in T5 were downregulated. These results align with findings from Aktas *et al*., who reported 41 transcripts affected by prolonged CIT, including genes involved in cell cycle regulation, apoptosis, stress responses, and cancer progression (10). Although this number seems modest, it might have significant implications for researchers focusing on specific genes, gene signatures, or pathways, particularly because these DEGs are closely linked to cancer development and progression. The broader impact of sample handling on the transcriptome varies widely across studies. von der Heyde *et al*. demonstrated that cold

ischemia times exceeding 15 minutes significantly impact the expression of mRNAs, proteins, and phosphosites in tumor and normal tissues across colorectal cancer, hepatocellular carcinoma, lung adenocarcinoma, and lung squamous cell carcinoma. The authors conclude that ischemia time is a crucial quality parameter for tissue collections used for target discovery and validation in cancer research (37). Using microarrays, Musella *et al*. found minimal effects, detecting only 0.2% DEGs in normal colon tissue and colorectal carcinoma within 6 hours, though two identified DEGs were oncogenes (17). In contrast, Spruessel *et al*. applying the same genotyping technique, reported significant changes, with 20% of detectable genes and proteins altered within 30 minutes post-excision (1). Grizzle *et al.* noted that while most studies found only 1–3% of transcripts affected, they often overlooked the functional impact of these genes (35). Our findings underscore that while RNA integrity remains stable within the first 30 minutes, transcriptional changes begin to emerge beyond this point. Even within a 60-minute CIT window, the occurring transcriptional changes can potentially alter the results of studies exploring cancer and adjacent tissue genetics. These observations highlight the importance of rapid tissue processing for accurate transcriptomic analyses in cancer research.

Our analysis focused on genes involved in cancer development and progression, as they are crucial for the studies of tumor margins and tissues adjacent to the tumor, where changes in the transcriptome may be subtle (21). The results demonstrate the impact of hypoxia on EMT, a process crucial for embryonic development and tissue regeneration, which is aberrantly reactivated in cancer to drive progression and metastasis through enhanced migration, invasiveness, stemness, and resistance to therapies (38). Genes such as SNAI, CDH1, MMP9, and S100A8, which play critical roles in the EMT in cancer, exhibited significantly altered

expression levels (adjusted p-value < 0.05) in our analysis. Snail (SNAI1) and Slug (SNAI2) are transcription factors known to bind to the promoter of CDH1, encoding E-cadherin, to repress its transcription (39). The suppression of CDH1, regulated by SNAI, is a key driver of EMT, contributing to cancer progression by promoting invasion and metastasis, and is recognized as a marker of malignancy and poor clinical prognosis (40). In our study, prolonged CIT caused significant alterations in the expression of SNAI and CDH1, with upregulation of SNAI and downregulation of CDH1. Moreover, the observed upregulation of MMP9, likely induced by CIT, may reflect further malignant changes as MMP family members facilitate cancer cell invasion and metastasis (38). These changes, often linked to malignancy, may be misinterpreted as early indicators of tumor progression in research on adjacent tissues. However, our findings suggest that these alterations result from prolonged CIT.

To gain an insight into complex biological mechanisms affected by CIT, we performed enrichment analysis of DEGs using two complementary approaches: a direct analysis of all DEGs and a WGCNA-based analysis of the two resulting modules, both evaluated through GO and KEGG. The direct DEG analysis provided a broad view of the pathways enriched across all DEGs, whereas WGCNA highlighted context-specific pathways within co-regulated gene modules.

GO and KEGG analyses of all DEGs identified malignancy-associated pathways, including apoptosis, ABC transporters, and TNF signaling, as well as stress response pathways (S4 Table). In comparison, other transcriptomic studies identified alteration of pathways linked to apoptosis, stress responses, cell cycle regulation, and cancer progression (10), as well as those associated with stimulus response and signal transduction (34). Bray *et al*. found no consistent gene ontology or pathway among 168 transcripts altered within 120 minutes of

CIT, highlighting a complex cellular response (16). In our study, despite targeting over 600 cancer-related genes, pathway enrichment was broad, supporting Bray *et al*.'s conclusions (S4 Table).

WGCNA identified two significantly downregulated co-expressed gene modules, ME2 and ME4, with distinct functional patterns (S7 Table). ME2 was primarily linked to apoptosis, with GO highlighting pathways like the execution phase of apoptosis and extrinsic apoptotic signaling, while KEGG linked it to FoxO, Hippo, and p53 signaling pathways. These findings underscore CIT's significant impact on apoptosis-related pathways within 60 minutes, potentially confounding studies that do not account for its effects. ME4 was enriched for metabolic pathways, including catabolic processes, homeostasis, pyruvate metabolism (GO), glycolysis/gluconeogenesis, and choline metabolism in cancer (KEGG). Both modules featured HIF-1 and cancer-related pathways, illustrating CIT’s dual impact on apoptosis and metabolism, complicating transcriptomic interpretations. These findings align with reports of ischemia-induced changes in CRC, affecting oncogenes and histone-related genes involved in nucleosome organization, cell cycle, DNA replication, and p53 signaling, beginning at 8–10 minutes (1) and persisting up to 60 minutes (17). Notably, ME4 exhibited minimal differences between T4 and T5, whereas ME2 showed a more pronounced variation between these time points, suggesting a slower reaction to CIT (Fig. 2A).

Our study highlights that beyond RNA degradation, ischemic stress actively alters gene expression, emphasizing the need to standardize CIT in human tissue collection. The consistency of our observations suggests these transcriptomic changes stem from surgical and tissue handling procedures rather than malignancy. A key strength of our study is the use of NGS RNA-seq, which offers greater sensitivity and accuracy than microarrays (11,17),

enabling robust detection of gene expression changes, even at low levels. Unlike previous studies, our approach analyzes over 600 genes, allowing a broader transcriptomic assessment. However, limitations include a small sample size and potential influences from enzymatic activity, warm ischemia, and other preanalytical factors (2,41). Additionally, our analysis used a targeted cancer-related gene panel, which does not capture all transcriptomic changes.

Conclusion

Our study highlights the significant impact of CIT on the transcriptomic profile of healthy colon mucosa, with a particular focus on cancer-related genes. Using targeted RNA sequencing and robust analyses, we identified differentially expressed genes primarily associated with apoptosis, hypoxia, and EMT, as well as pathways involved in cancer development and progression. Our findings underscore the importance of considering CIT as a critical preanalytical variable, as prolonged ischemia can alter gene expression in ways that may mimic malignant changes, leading to misinterpretations of results and incorrect clinical conclusions. Importantly, our results suggest that CIT should be kept below 45 minutes to minimize its impact on transcriptomic profiles. However, as the effects of CIT might be tissue-dependent, this recommendation should be applied with caution when extrapolating to other tissues.

Acknowledgements

We thank all the anonymous patients for acceptance to participate, sample contribution and information provided in the questionnaire. We also thank surgeons, and nurses involved in the patient recruitment process, collaborating technicians, diagnosticians and pathologists from University Clinical Centre in Gdańsk, University Hospital in Cracow and Specialist

Hospital in Kościerzyna. This study was sponsored by the Foundation for Polish Science (FNP) under the International Research Agendas Program to J.P.D. and A.P., co-financed by the European Union under the European Regional Development Fund.

References

1. Spruessel A, Steimann G, Jung M, Lee SA, Carr T, Fentz AK, et al. Tissue ischemia time affects gene and protein expression patterns within minutes following surgical tumor excision. Biotechniques [Internet]. 2004 [cited 2024 Oct 15];36(6). Available from: https://www.tandfonline.com/action/journalInformation?journalCode=ibtn20
2. Galissier T, Schneider C, Nasri S, Kanagaratnam L, Fichel C, Coquelet C, et al. Biobanking of Fresh-Frozen Human Adenocarcinomatous and Normal Colon Tissues: Which Parameters Influence RNA Quality? PLoS One. 2016 Apr 28;11(4):e0154326.
3. Conroy MC, Lacey B, Bešević J, Omiyale W, Feng Q, Effingham M, et al. UK Biobank: a globally important resource for cancer research. Vol. 128, British Journal of Cancer. Springer Nature; 2023. p. 519–27.
4. Filipowicz N, Drężek K, Horbacz M, Wojdak A, Szymanowski J, Rychlicka-Buniowska E, et al. Comprehensive cancer-oriented biobanking resource of human samples for studies of post-zygotic genetic variation involved in cancer predisposition. PLoS One. 2022 Apr 1;17(4 April).
5. Bonizzi G, Zattoni L, Capra M, Cassi C, Taliento G, Ivanova M, et al. Standard operating procedures for biobank in oncology. Front Mol Biosci. 2022 Aug 26;9.
6. Foley ME, Vinson AJ, Skinner TAA, Kiberd BA, Tennankore KK. The Impact of Combined Warm and Cold Ischemia Time on Post-transplant Outcomes. Can J Kidney Health Dis. 2023 Jan 1;10.
7. Pedersen IS, Thomassen M, Tan Q, Kruse T, Thorlacius-Ussing O, Garne JP, et al. Differential effect of surgical manipulation on gene expression in normal breast tissue and breast tumor tissue. Molecular Medicine. 2018 Dec 16;24(1):57.
8. Ma Y, Dai H, Kong X. Impact of warm ischemia on gene expression analysis in surgically removed biosamples. Anal Biochem. 2012 Apr;423(2):229–35.
9. Shabihkhani M, Lucey GM, Wei B, Mareninov S, Lou JJ, Vinters H V., et al. The procurement, storage, and quality assurance of frozen blood and tissue biospecimens in pathology, biorepository, and biobank settings. Clin Biochem. 2014 Mar;47(4–5):258–66.
10. Aktas B, Sun H, Yao H, Shi W, Hubbard R, Zhang Y, et al. Global gene expression changes induced by prolonged cold ischemic stress and preservation method of breast cancer tissue. Mol Oncol. 2014 May 17;8(3):717–27.
11. Liu NW, Sanford T, Srinivasan R, Liu JL, Khurana K, Aprelikova O, et al. Impact of Ischemia and Procurement Conditions on Gene Expression in Renal Cell Carcinoma. Clinical Cancer Research. 2013 Jan 1;19(1):42–9.
12. Bao WG, Zhang X, Zhang JG, Zhou WJ, Bi TN, Wang JC, et al. Biobanking of Fresh-frozen Human Colon Tissues: Impact of Tissue Ex-vivo Ischemia Times and Storage Periods on RNA Quality. Ann Surg Oncol. 2013 May 19;20(5):1737–44.
13. Micke P, Ohshima M, Tahmasebpoor S, Ren ZP, Östman A, Pontén F, et al. Biobanking of fresh frozen tissue: RNA is stable in nonfixed surgical specimens. Laboratory Investigation. 2006 Feb;86(2):202–11.

14. Viana CR, Neto CS, Kerr LM, Palmero EI, Marques MMC, Colaiacovo T, et al. The interference of cold ischemia time in the quality of total RNA from frozen tumor samples. Cell Tissue Bank. 2013 Jun;14(2):167–73.
15. Zeugner S, Mayr T, Zietz C, Aust DE, Baretton GB. RNA quality in fresh-frozen gastrointestinal tumor Specimens—Experiences from the tumor and healthy tissue bank TU dresden. Recent Results in Cancer Research. 2015;199:85–93.
16. Bray SE, Paulin FEM, Fong SC, Baker L, Carey FA, Levison DA, et al. Gene expression in colorectal neoplasia: Modifications induced by tissue ischaemic time and tissue handling protocol. Histopathology. 2010 Jan;56(2):240–50.
17. Musella V, Verderio P, Reid JF, Pizzamiglio S, Gariboldi M, Callari M, et al. Effects of Warm Ischemic Time on Gene Expression Profiling in Colorectal Cancer Tissues and Normal Mucosa. PLoS One. 2013 Jan 7;8(1):e53406.
18. Zhou L, Xu G, Huang F, Chen W, Zhang J, Tang Y. Apoptosis related genes mediated molecular subtypes depict the hallmarks of the tumor microenvironment and guide immunotherapy in bladder cancer. BMC Med Genomics. 2023 Dec 1;16(1).
19. Li D, Li L, Yang W, Chen L, Chen X, Wang Q, et al. Prognostic values of SNAI family members in breast cancer patients. Ann Transl Med. 2020 Aug;8(15):922–922.
20. Van 't Veer LJ, Bernards R. Enabling personalized cancer medicine through analysis of gene-expression patterns. Vol. 452, Nature. Nature Publishing Group; 2008. p. 564–70.
21. Andreou M, Jąkalski M, Duzowska K, Filipowicz N, Kostecka A, Davies H, et al. Prelude to malignancy: A gene expression signature in normal mammary gland from breast cancer patients suggests pre-tumorous alterations and is associated with adverse outcomes. Int J Cancer. 2024 Nov 8;155(9):1616–28.
22. Mieczkowska A, Schumacher A, Filipowicz N, Wardowska A, Zieliński M, Madanecki P, et al. Immunophenotyping and transcriptional profiling of in vitro cultured human adipose tissue derived stem cells. Sci Rep. 2018 Jul 27;8(1):11339.
23. Matsubara T, Soh J, Morita M, Uwabo T, Tomida S, Fujiwara T, et al. DV200 Index for Assessing RNA Integrity in Next-Generation Sequencing. Biomed Res Int. 2020 Jan 27;2020(1).
24. Andreou M, Jąkalski M, Duzowska K, Filipowicz N, Kostecka A, Davies H, et al. Prelude to malignancy: A gene expression signature in normal mammary gland from breast cancer patients suggests pre-tumorous alterations and is associated with adverse outcomes. Int J Cancer. 2024 Nov 8;155(9):1616–28.
25. Dobin A, Davis CA, Schlesinger F, Drenkow J, Zaleski C, Jha S, et al. STAR: ultrafast universal RNA-seq aligner. Bioinformatics. 2013 Jan 1;29(1):15–21.
26. Robinson MD, Oshlack A. A scaling normalization method for differential expression analysis of RNA-seq data. Genome Biol. 2010 Mar 2;11(3):R25.
27. Lê S, Josse J, Husson F. **FactoMineR** : An *R* Package for Multivariate Analysis. J Stat Softw. 2008;25(1).
28. Zhang Y, Parmigiani G, Johnson WE. ComBat-seq: batch effect adjustment for RNA-seq count data. NAR Genom Bioinform. 2020 Sep 1;2(3).
29. Langfelder P, Horvath S. WGCNA: an R package for weighted correlation network analysis. BMC Bioinformatics. 2008 Dec 29;9(1):559.
30. Ritchie ME, Phipson B, Wu D, Hu Y, Law CW, Shi W, et al. limma powers differential expression analyses for RNA-sequencing and microarray studies. Nucleic Acids Res. 2015 Apr 20;43(7):e47–e47.

31. Liao Y, Wang J, Jaehnig EJ, Shi Z, Zhang B. WebGestalt 2019: gene set analysis toolkit with revamped UIs and APIs. Nucleic Acids Res. 2019 Jul 2;47(W1):W199–205.
32. Matsubara T, Soh J, Morita M, Uwabo T, Tomida S, Fujiwara T, et al. DV200 Index for Assessing RNA Integrity in Next-Generation Sequencing. Biomed Res Int. 2020 Jan 27;2020(1).
33. Vasaikar S V., Deshmukh AP, den Hollander P, Addanki S, Kuburich NA, Kudaravalli S, et al. EMTome: a resource for pan-cancer analysis of epithelial-mesenchymal transition genes and signatures. Br J Cancer. 2021 Jan 5;124(1):259–69.
34. Borgan E, Navon R, Vollan HKM, Schlichting E, Sauer T, Yakhini Z, et al. Ischemia caused by time to freezing induces systematic microRNA and mRNA responses in cancer tissue. Mol Oncol. 2011 Dec 29;5(6):564–76.
35. Grizzle WE, Otali D, Sexton KC, Atherton DS. Effects of Cold Ischemia on Gene Expression: A Review and Commentary. Biopreserv Biobank. 2016 Dec;14(6):548–58.
36. Heumüller-Klug S. Degradation of intestinal mRNA: A matter of treatment. World J Gastroenterol. 2015;21(12):3499.
37. von der Heyde S, Raman N, Gabelia N, Matias-Guiu X, Yoshino T, Tsukada Y, et al. Tumor specimen cold ischemia time impacts molecular cancer drug target discovery. Cell Death Dis. 2024 Sep 26;15(9):691.
38. Huang Y, Hong W, Wei X. The molecular mechanisms and therapeutic strategies of EMT in tumor progression and metastasis. Vol. 15, Journal of Hematology and Oncology. BioMed Central Ltd; 2022.
39. Tam SY, Wu VWC, Law HKW. Hypoxia-Induced Epithelial-Mesenchymal Transition in Cancers: HIF-1α and Beyond. Front Oncol. 2020 Apr 8;10.
40. Cano A, Pérez-Moreno MA, Rodrigo I, Locascio A, Blanco MJ, del Barrio MG, et al. The transcription factor Snail controls epithelial–mesenchymal transitions by repressing E-cadherin expression. Nat Cell Biol. 2000 Feb 13;2(2):76–83.
41. Medeiros F, Rigl CT, Anderson GG, Becker SH, Halling KC. Tissue Handling for Genome-Wide Expression Analysis: A Review of the Issues, Evidence, and Opportunities. Arch Pathol Lab Med. 2007 Dec 1;131(12):1805–16.

Supporting information

S1 Table. Pseudonymized list of patients that were included in the study with the information on gender, ICD10 (International Statistical Classification of Diseases and Related Health Problems, 10th Revision), sample time points, type of tissue, and RNA Integrity Number (RIN) values. (XLSX)

S2 Table. Target genes included in the designed panel and functional classification of particular transcripts. (a) Gene symbols of targets included in the panel, (b) Ensembl ID, (c)

Ensembl ID as in Gencode (version 35), (d) Gene symbol according to the HUGO Gene Nomenclature Committee, (e) EntrezID, (f) Full name of the gene, (g) - (p) Functional classification. **(XLSX)**

S3 Table. Differentially Expressed Genes (DEGs) between time points T5 (60 minutes) and T0 (0 minutes). (a) Ensembl ID as in Gencode (version 35), (b) Gene symbol, (c) Direction of the expression change: up - upregulation, down - downregulation, (d) logFoldChange (logFC), (e) p-value, (f) False Discovery Rate (FDR), (g) Indication if the gene was included in our panel: N - no, Y - yes, (h) Information regarding the comparison between different groups; T5 (time point 5, 60 minutes after resection), T4 (time point 4, 45 minutes after resection) and T0 (time point 0, 0 minutes after resection). **(XLSX)**

S4 Table. The results of enrichment analysis for differentially expressed genes (DEGs) between T5 (time point 5, 60 minutes after resection) and T0 (time point 0, 0 minutes after resection). (a) Gene Ontology Term ID and Kyoto Encyclopedia of Genes and Genomes (KEGG) ID, (b) short description of identified Gene Ontology (GO) terms, (c) p-value, (d) False Discovery Rate (FDR), (e) ratio of genes from our targeted panel vs. all genes associated with the particular GO term, (f) genes from our analysis associated with the particular GO term, (g) information regarding the comparison between different groups; T5 (time point 5, 60 minutes after resection) and T0 (time point 0, 0 minutes after resection). **(XLSX)**

S5 Table. Genes included in weighted gene co-expression network analysis (WCGNA) module eigengene 2 (ME2). (a) gene symbol, (b) description, (c) functional classification. **(XLSX)**

S6 Table. Genes included in weighted gene co-expression network analysis (WCGNA) module eigengene 4 (ME4). (a) gene symbol, (b) description, (c) functional classification. **(XLSX)**

S7 Table. Gene Ontology (GO) terms and KEGG pathways identified via enrichment analyses, including cluster membership information. Enrichment analysis was conducted for genes from weighted gene co-expression network analysis (WCGNA) from statistically significant modules - ME2 and ME4. (a) Gene Ontology Term ID and Kyoto Encyclopedia of Genes and Genomes (KEGG) ID, (b) short description of identified Gene Ontology (GO) terms,

(c) p-value, (d) False Discovery Rate (FDR), (e) ratio of genes included in the panel, associated with the particular GO term, (f) Genes associated with the particular GO term, (g) Information regarding the comparison between different groups: T5 (time point 5, 60 minutes after resection) and T0 (time point 0, 0 minutes after resection). **(XLSX)**

PONE-D-25-11860

The effect of cold ischemia time on hypoxia, EMT, and apoptosis pathways in normal colon mucosa

Katarzyna Duzowska, Mikołaj Opiełka, Kinga Drężek-Chyła, Anna Kostecka, Monika Horbacz, Jarosław Skokowski, Olga Rostkowska, Jarosław Kobiela, Leszek Kalinowski, Jan P. Dumanski, Arkadiusz Piotrowski, Marcin Jąkalski, Natalia Filipowicz

Comments to the Author (Reviews provided by Dr. Yingkun Xu on October, 17, 2025)

The answers are marked in green color.

1. Is the manuscript technically sound, and does the data support the conclusions?

The manuscript must describe a technically sound piece of scientific research with data that supports the conclusions. Experiments must have been conducted rigorously, with appropriate controls, replication, and sample sizes. The conclusions must be drawn appropriately based on the data presented.

Reviewer #1: Yes

Reviewer #2: Yes

2. Has the statistical analysis been performed appropriately and rigorously?

Reviewer #1: Yes

Reviewer #2: Yes

3. Have the authors made all data underlying the findings in their manuscript fully available?

The PLOS Data policy requires authors to make all data underlying the findings described in their manuscript fully available without restriction, with rare exception (please refer to the Data Availability Statement in the manuscript PDF file). The data should be provided as part of the manuscript or its supporting information, or deposited to a public repository. For example, in addition to summary statistics, the data points behind means, medians and variance measures should be available. If there are restrictions on publicly sharing data—e.g. participant privacy or use of data from a third party—those must be specified.

Reviewer #1: Yes

Reviewer #2: Yes

4. Is the manuscript presented in an intelligible fashion and written in standard English?

PLOS ONE does not copyedit accepted manuscripts, so the language in submitted articles must be clear, correct, and unambiguous. Any typographical or grammatical errors should be corrected at revision, so please note any specific errors here.

Reviewer #1: Yes

Reviewer #2: Yes

5. Review Comments to the Author

Please use the space provided to explain your answers to the questions above. You may also include additional comments for the author, including concerns about dual publication,

research ethics, or publication ethics. (Please upload your review as an attachment if it exceeds 20,000 characters)

Reviewer #1: accepted.

explain your answers to the questions above. You may also include additional comments for the author, including concerns about dual publication, research ethics, or publication ethics. (Please upload your review as an attachment if it exceeds 20,000 characters) (Limit 200 to 20000 Characters)

Reviewer #2: The paper explains the effect of cold ischemia time (CIT) and its importance in transcriptomic studies and reproducibility. The study objective evaluating CIT effects on cancer-related genes is relevant and practical. The findings from this study suggest a CIT limit (≤45–60 min), information that is valuable for the research community.

The number of samples, patient count and CIT intervals are clearly described but whether all 54 samples were paired per patient across all time points needs further clarification.

Answer: All 54 samples used in our study originate from 9 CRC patients, with samples collected at 6 distinct time points as defined in the manuscript (9 patients × 6 time points = 54 samples). Thus, each patient contributed one sample per time point, resulting in a fully paired dataset.

We accounted for the paired nature of the RNAseq data by treating the patient ID as batch and removing its effect from the data with ComBat-seq. The sentence clarifying this was modified in lines 173-175.

Overall, the analysis is comprehensive. The combination of RNA-seq, functional enrichment and WGCNA strengthens the biological interpretation. Identifying DEGs and linking them to apoptosis, hypoxia, EMT and cancer-related pathways is relevant and informative.

6. PLOS authors have the option to publish the peer review history of their article (what does this mean?). If published, this will include your full peer review and any attached files.

If you choose “no”, your identity will remain anonymous but your review may still be made public.

Do you want your identity to be public for this peer review? For information about this choice, including consent withdrawal, please see our Privacy Policy.

Reviewer #1: No

Reviewer #2: No
