# Supplementary material for: The effect of cold ischemia time on hypoxia, EMT, and apoptosis pathways in normal colon mucosa
Source: PLoS One. 2026 Mar 20;21(3):e0321432. doi: 10.1371/journal.pone.0321432 (PMC13004397; doi:10.1371/journal.pone.0321432)
Supplement: Attachment — Submitted filename: PONED2511860_Responses_to_Reviewers.docx [file pone.0321432.s004.docx]

**PONE-D-25-11860**

**The effect of cold ischemia time on hypoxia, EMT, and apoptosis pathways in normal colon mucosa**

Katarzyna Duzowska, Mikołaj Opiełka, Kinga Drężek-Chyła, Anna Kostecka, Monika Horbacz, Jarosław Skokowski, Olga Rostkowska, Jarosław Kobiela, Leszek Kalinowski, Jan P. Dumanski, Arkadiusz Piotrowski, Marcin Jąkalski, Natalia Filipowicz

***Comments to the Author (Reviews provided by Dr. Yingkun Xu on October, 17, 2025)***

***The answers are marked in green color.***

*1. Is the manuscript technically sound, and does the data support the conclusions?*

*The manuscript must describe a technically sound piece of scientific research with data that supports the conclusions. Experiments must have been conducted rigorously, with appropriate controls, replication, and sample sizes. The conclusions must be drawn appropriately based on the data presented.*

*Reviewer #1: Yes*

*Reviewer #2: Yes*

*2. Has the statistical analysis been performed appropriately and rigorously?*

*Reviewer #1: Yes*

*Reviewer #2: Yes*

*3. Have the authors made all data underlying the findings in their manuscript fully available?*

*The PLOS Data policy requires authors to make all data underlying the findings described in their manuscript fully available without restriction, with rare exception (please refer to the Data Availability Statement in the manuscript PDF file). The data should be provided as part of the manuscript or its supporting information, or deposited to a public repository. For example, in addition to summary statistics, the data points behind means, medians and variance measures should be available. If there are restrictions on publicly sharing data—e.g. participant privacy or use of data from a third party—those must be specified.*

*Reviewer #1: Yes*

*Reviewer #2: Yes*

*4. Is the manuscript presented in an intelligible fashion and written in standard English?*

*PLOS ONE does not copyedit accepted manuscripts, so the language in submitted articles must be clear, correct, and unambiguous. Any typographical or grammatical errors should be corrected at revision, so please note any specific errors here.*

*Reviewer #1: Yes*

*Reviewer #2: Yes*

*5. Review Comments to the Author*

*Please use the space provided to explain your answers to the questions above. You may also include additional comments for the author, including concerns about dual publication, research ethics, or publication ethics. (Please upload your review as an attachment if it exceeds 20,000 characters)*

*Reviewer #1: accepted.*

*explain your answers to the questions above. You may also include additional comments for the author, including concerns about dual publication, research ethics, or publication ethics. (Please upload your review as an attachment if it exceeds 20,000 characters) (Limit 200 to 20000 Characters)*

*Reviewer #2: The paper explains the effect of cold ischemia time (CIT) and its importance in transcriptomic studies and reproducibility. The study objective evaluating CIT effects on cancer-related genes is relevant and practical. The findings from this study suggest a CIT limit (≤45–60 min), information that is valuable for the research community.*

***The number of samples, patient count and CIT intervals are clearly described but whether all 54 samples were paired per patient across all time points needs further clarification.***

**Answer:** All 54 samples used in our study originate from 9 CRC patients, with samples collected at 6 distinct time points as defined in the manuscript (9 patients × 6 time points = 54 samples). Thus, each patient contributed one sample per time point, resulting in a fully paired dataset.

We accounted for the paired nature of the RNAseq data by treating the patient ID as batch and removing its effect from the data with ComBat-seq. The sentence clarifying this was modified in lines 173-175.

*Overall, the analysis is comprehensive. The combination of RNA-seq, functional enrichment and WGCNA strengthens the biological interpretation. Identifying DEGs and linking them to apoptosis, hypoxia, EMT and cancer-related pathways is relevant and informative.*

*6. PLOS authors have the option to publish the peer review history of their article (what does this mean?). If published, this will include your full peer review and any attached files.*

*If you choose “no”, your identity will remain anonymous but your review may still be made public.*

*Do you want your identity to be public for this peer review? For information about this choice, including consent withdrawal, please see our Privacy Policy.*

*Reviewer #1: No*

*Reviewer #2: No*
