# Supplementary material for: The effect of cold ischemia time on hypoxia, EMT, and apoptosis pathways in normal colon mucosa
Source: PLoS One. 2026 Mar 20;21(3):e0321432. doi: 10.1371/journal.pone.0321432 (PMC13004397; doi:10.1371/journal.pone.0321432)
Supplement: Attachment — Submitted filename: 20260121_PONED2511860_responses_to_reviewers.docx [file pone.0321432.s005.docx]

**PONE-D-25-11860R1 “The effect of cold ischemia time on hypoxia, EMT, and apoptosis pathways in normal colon mucosa”**

**Answers to the comments:**

**Comment 1:**

The proposed “safe CIT threshold” appears overly optimistic and contradicts multiple prior studies. The manuscript recommends limiting CIT to 45–60 minutes to minimize molecular artifacts. However, previous literature shows that both gene and protein expression in colonic tissue can be significantly altered within minutes.

• Spruessel et al. demonstrated that a few minutes to 30 minutes of ischemia can markedly change gene and protein expression in colon samples: <https://pubmed.ncbi.nlm.nih.gov/15211754/>

• von der Heyde et al. (2024, Cell Death & Disease, Nature Publishing Group) showed that proteomic dysregulation emerges above 15 minutes of ischemia:

“Specimen ischemia time above 15 min is mostly associated with a dysregulation of proteins in the immune-response pathway.”

<https://www.nature.com/articles/s41419-024-07090-x>

These findings suggest that the authors’ proposed CIT tolerance window may not be sufficiently conservative.

**Answer 1:**

Thank you for this valuable comment.

Our original recommendation of a 45–60 minutes CIT threshold was based strictly on the results obtained in the present study, which employed a targeted transcriptomic approach covering 634 genes primarily involved in apoptosis, cell death, hypoxia, epithelial‑to‑mesenchymal transition (EMT), and cancer‑related pathways. Within this restricted gene set and thorough analytical framework, we did not observe statistically significant transcriptional changes within the first 30 minutes post‑excision. The earliest detectable alterations were observed between 30 and 45 minutes, with a substantially larger number of differentially expressed genes emerging after 45 minutes.

After careful consideration of the reviewer’s comment and the cited literature, we agree that a more conservative recommendation is warranted. We have therefore **revised the manuscript to propose a stricter CIT threshold of ≤30 minutes**, which better aligns with existing evidence and reflects a precautionary approach. Corresponding changes have been made in the manuscript (lines 50 and 402).

We also acknowledge that our findings may appear contradictory to several previous studies on CIT. This discrepancy is already discussed in the Discussion section, where we cite selection of articles on CIT (lines 304-334) including Spruessel et al. and von der Heyde et al. (2024). We now modified slightly this section by adding information from von der Heyde et al. on ischemia time being more pronounced at the proteomic and phosphoproteomic levels than at the mRNA level (lines 319-321). We would also like to stress that we do not claim in our paper that the CIT threshold guidelines would be applicable to all kinds of studies, including proteomics.

We believe that the revised recommendation of a ≤30‑minute CIT threshold, along with a clearer contextualization of our results to other transcriptomic and proteomic studies, strengthens the manuscript.

**Comment 2:**

The study relies solely on mRNA measurements and lacks multi-omic validation. No proteomic, immunohistochemical, or additional orthogonal assays are provided. This is a notable gap because proteomic alterations often occur earlier and more sensitively than transcriptomic changes.

For example, von der Heyde et al. (2024) report: “The proteomics analysis revealed that specimen ischemia time above 15 min is mostly associated with a dysregulation of proteins in the immune-response pathway. ”https://www.nature.com/articles/s41419-024-07090-x

Without multi-omics confirmation, it is difficult to determine whether the reported mRNA changes reflect true biological responses or early degradation phenomena.

**Answer 2:**

We appreciate this thoughtful comment highlighting the importance of multi-omic approaches in assessing tissue quality and preanalytical variables. We agree that proteomic and other assays can offer complementary insights, particularly regarding early ischemia-related changes that may not be immediately reflected at the transcript level. However, our study was intentionally focused on transcriptomic profiling, using a targeted panel of 634 genes related to apoptosis, hypoxia, cell death, and cancer progression. This approach, although narrower than full transcriptome or proteome analysis, was chosen for its clinical relevance and feasibility in standardizing preanalytical workflows. Due to technical and resource constraints, multi-omic validation was beyond the scope of the current study. We believe that our results offer a practical, transcriptome-focused reference point that can guide tissue handling protocols and inform future integrative studies. To stress limitation of our approach we added additional sentence in the Conclusion section (lines 405-407).

**Comment 3:**

Warm ischemia time (WIT) is acknowledged but not recorded or modeled, which is inconsistent with established standards. Although the authors state that WIT is important, they do not:

• document each patient’s WIT;

• include WIT as a covariate in statistical models (e.g., edgeR, linear models);

• analyze interactions between WIT and CIT.

This is problematic because prior work has emphasized that WIT must be explicitly accounted for. Musella et al. (2013) show that both WIT and CIT shape molecular stability in colon tissue: “A critical time point for tissue handling in colon seems to be 60 minutes at room temperature … accounting for warm ischemia in this tumor type.”

https://journals.plos.org/plosone/article?id=10.1371/journal.pone.0053406
Without controlling for WIT, the attribution of transcriptional changes solely to CIT is not well supported.

**Answer 3:**

Thank you very much for this important comment. We fully agree that warm ischemia time (WIT) is a critical preanalytical factor and appreciate the opportunity to clarify our rationale and study limitations regarding this aspect.

As noted in the introduction (lines 56–70), we acknowledge the impact of WIT on gene expression and reference prior studies addressing its role. In our setting, WIT could not be precisely measured for each patient due to the clinical priority of ensuring a safe and effective surgical outcome. WIT, defined as the period during which the tissue remains in the body without effective oxygen supply, is inherently difficult to standardize and control, especially during open surgical procedures.

In case of our cohort all the procedures were performed using an open surgical approach, which minimizes warm ischemia time (WIT) by allowing immediate specimen extraction following devascularization. In contrast, laparoscopic and robotic approaches require placement of the devascularized specimen into an extraction bag, with removal occurring only at the completion of the procedure. In the present case series, all operations consisted of left-sided colectomies or rectal resections, with operative times ranging from 1 hour 15 minutes to 3 hours 20 minutes. Considering the open approach and the review specific technical aspects of the procedures, it is reasonable to assume that **WIT did not exceed 40 minutes in any case**. This variable should be considered unmodifiable, as it is largely determined by intra-abdominal anatomical conditions.

We respectfully note that although we cited Musella et al. (2013) to highlight preanalytical variability, the ischemia time discussed in their study appears to align more closely with cold ischemia time (CIT), not WIT. In the methods section, they state that “the time was measured starting from patient’s surgical excision and the first timepoint analysed (T0)”, indicating that their 60-minute measurement refers to *ex-vivo* conditions.

Taking all of these into account we recognized the importance of addressing this limitation in a transparent way. We have now added a suitable statement regarding WIT in the Materials and Methods: “Study Design and Sample Handling” (lines 116-124) emphasizing the use of open surgery, estimated upper limit of WIT and difficulty recording patient-specific WIT under current surgical protocols.

We hope this provides clarity and adequately addresses the reviewer’s concern.
